# Supplementary material for: The Synthesis and Antibacterial Properties of Pillar[5]arene with Streptocide Fragments
Source: Pharmaceutics. 2023 Nov 23;15(12):2660. doi: 10.3390/pharmaceutics15122660 (PMC10747162; doi:10.3390/pharmaceutics15122660)
Supplement: Supplementary file 1 [file pharmaceutics-15-02660-s001.zip › pharmaceutics-2686808-supplementary.pdf]

Electronic Supplementary Information (21 pages)

1. Materials and methods 2

2. NMR, MALDI TOF MS, IR spectra of compounds 2-9 4

3. Dynamic light scattering 20

4. References 21

1. Materials and methods

Pillar[5]arene **1** were synthesized according to the literature procedure S1.

Most chemicals were purchased from Aldrich and used as received without additional purification. Organic solvents were purified in accordance with standard procedures.

^{1}H NMR, ^{13}C NMR spectra were obtained on a Bruker Avance-400 spectrometer ($^{13}C\{^{1}H\}$ – 100 MHz and ^{1}H – 400 MHz). Chemical shifts were determined against the signals of residual protons of deuterated solvent (DMSO-d_6, D_2O, $CDCl_3$). The concentration of sample solutions was 5-10 mM.

Attenuated total internal reflectance IR spectra were recorded with Spectrum 400 (Perkin Elmer) Fourier spectrometer. The IR spectra from 4000 to 400 cm^{-1} were considered in this analysis. The spectra were measured with 1 cm^{-1} resolution and 64 scans co-addition.

Elemental analysis was performed with Perkin Elmer 2400 Series II instrument.

Mass spectra (MALDI-TOF) were recorded on an Ultraflex III mass spectrometer in a 4-nitroaniline matrix.

Electrospray ionization mass spectra (ESI) were obtained on an AmazonX mass spectrometer (Bruker Daltonik GmbH, Bremen, Germany). The measurements were carried out in the regime of positive ions registration in the range of *m/z* from 100 to 2800. The voltage on the capillary was - 4500 V. Nitrogen was used as the gas-drier with a temperature of 300 °C and a flow rate of 10 $L{\cdot}min^{1}$. The compounds were dissolved in acetonitrile to a concentration of 10^{-6} g/L. Data was processed using DataAnalysis 4.0 (Bruker Daltonik GmbH, Bremen, Germany).

Additional control of the purity of compounds and monitoring of the reaction were carried out by thin-layer chromatography using Silica G, 200 μm plates, UV 254.

Dynamic light scattering (DLS). The particle size and zeta potential were determined by the Zetasizer Nano ZS instrument at 20 °C. The instrument contains 4mW He-Ne laser operating at a wave length of 633 nm and incorporated noninvasive backscatter optics (NIBS). The measurements were performed at the detection angle of 173° and the software automatically determined the measurement position within the quartz cuvette. The 1-1000 μM of **3-8** in water were prepared. Electrophoretic mobility of different samples was using a fold capillary cuvette (Folded Capillary Cell DTS1060, Malvern, U.K.). The experiments were carried out for each solution in triplicate.

Biological experiments.

Cytotoxicity. The ability of macrocyclic compounds **2**, **6** to inhibit the viability and proliferative activity of LEK cells and A549 cells was investigated using the MTT test according to S2. A549 – human alveolar adenocarcinoma cell line and bovine embryo lung epithelial (LEK) cells (both lines from Russian Collection of Cell Cultures of Vertebrates (CCCV)) were cultured in DMEM medium supplemented with 10% fetal bovine serum BioSera, 2 mM L-glutamine and 100 Units/ml of penicillin and streptomycin in a humid atmosphere with 5% CO_2 at 37 °C. Cells were seeded in 96-well plates at a concentration of 10^4 cells/well. After 24 h of cultivation, the medium was removed from the wells and replaced with a fresh one with the addition of the test substances. The volume of the culture medium in the wells was 100 μl. After 48 h incubation of cells in the presence of substances, the medium in the wells was replaced with a fresh one containing MTT reagent at a final concentration of 0.5 mg/ml. The cells were incubated with MTT for 3 h at 37 °C in an atmosphere of 5% CO_2. Then, the medium was aspirated from the wells and 100 μL of dimethyl sulfoxide was added thereto, after which plates were incubated at 37 °C for 15 min in the dark until the formazan crystals dissolve. The optical density of the formazan solution in the wells was measured using a plate reader (BioRad xMarkTM Microplate Spectrophotometer, USA) at a wavelength of 570 nm. Three series of experiments were carried out with 5 replicates for each variant in the series.

Biofilm prevention concentration evaluation. Cultures of *Staphylococcus aureus* and *Pseudomonas aeruginosa* were grown in BM-broth for 18 h to a density of approximately 1×10^{11}

cells/mL. Then, the cultures were diluted to 1×10^6 cells/mL and added to 24-well culture plates (SPL lifesciences) in a volume of 1 mL/well. Pillar[5]arenes were added to wells in the final concentrations range 5 μM – 70 μM and the plates were incubated under static conditions for 24 h at 37 °C. Biofilms formed after cultivation were washed with sodium phosphate buffer (pH = 7.4) from planktonic cells, dried under sterile conditions during 24 hours, and stained with 1 mL 0.1% crystal violet solution for 20 minutes. Stained biofilms were washed three times with sodium phosphate buffer (pH = 7.4) and dried for at least 30 min. 1 mL of 96% ethanol was added to resolubilize the crystal violet bound with biofilms, and the absorbance was measured at 570 nm with the BIO-Rad xMark Microplate spectrophotometer. Cell-free wells incubated with pure medium subjected to all staining manipulations were used as control. The concentration of macrocycle at which no biofilm development was detected was considered as BPC.

Minimal inhibitory concentration evaluation. The MIC of the macrocycles and sulfanilamide was determined by resazurin test in 96-well microtiter plates (SPL lifesciences) according to S3. The agents were subsequently diluted in L-broth (1:1). The concentration ranges were 1.2 μM - 300 μM for pillar[5]arenes and 19 μM - 4800 μM for sulfonamide. Then the bacterial suspensions (1×10^8 cells/mL final concentration) were added to wells and plates were incubated for 24 hours at 37 °C in humidified atmosphere. After incubation the resazurin solution (10 μL, 1 %) was added to wells and plates incubated for several hours at 37 °C. A change in color from purple to pink or discoloration was considered as evidence of the microorganisms' development in the well.

Ames test. The mutagenicity of macrocycles was assessed using the Ames test with Salmonella typhimurium TA98 and TA100 strains as described in S4.

Statistical analisys. All experiments were performed in biological triplicates (i.e. newly prepared cultures and medium) with at least three repeats in each run. The average values for the data groups and the confidence interval of the populations ($p \leq 0.05$) are calculated. Significance of group data differences were calculated in the variants without treatment using the nonparametric Mann-Whitney test.

2. NMR, MALDI TOF MS, IR spectra of compounds 2-9

Figure S1. ^{1}H NMR spectrum of 4,8,14,18,23,26,28,31,32,35-deca-[thioethane(2'-(N,N-dimethyl)amino)ethoxy]-pillar[5]arene **(2)**. $CDCl_3$, 298 K, 400 MHz.

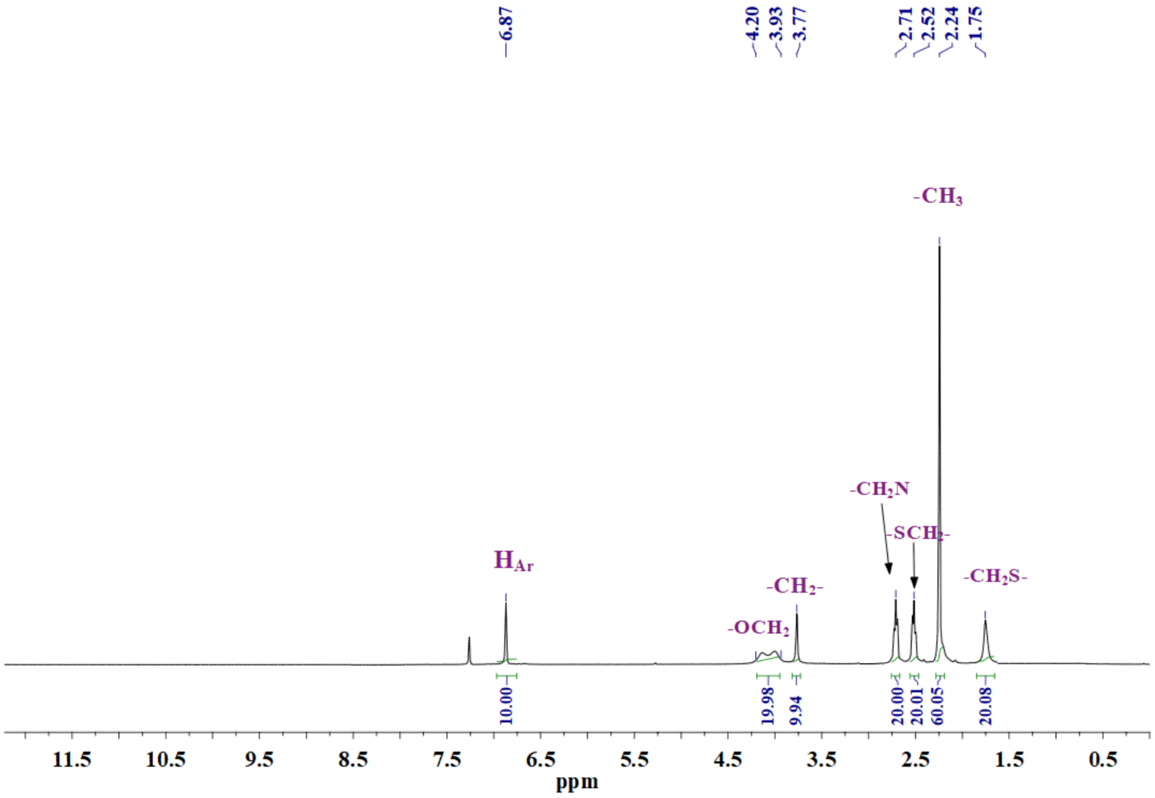

Figure S2. ^{1}H NMR spectrum of 4,8,14,18,23,26,28,31,32,35-deca-[thioethane(2'-(N,N,N-trimethyl)ammonium)ethoxy]-pillar[5]arene iodide **(3)**. DMSO-d_6, 298 K, 400 MHz.

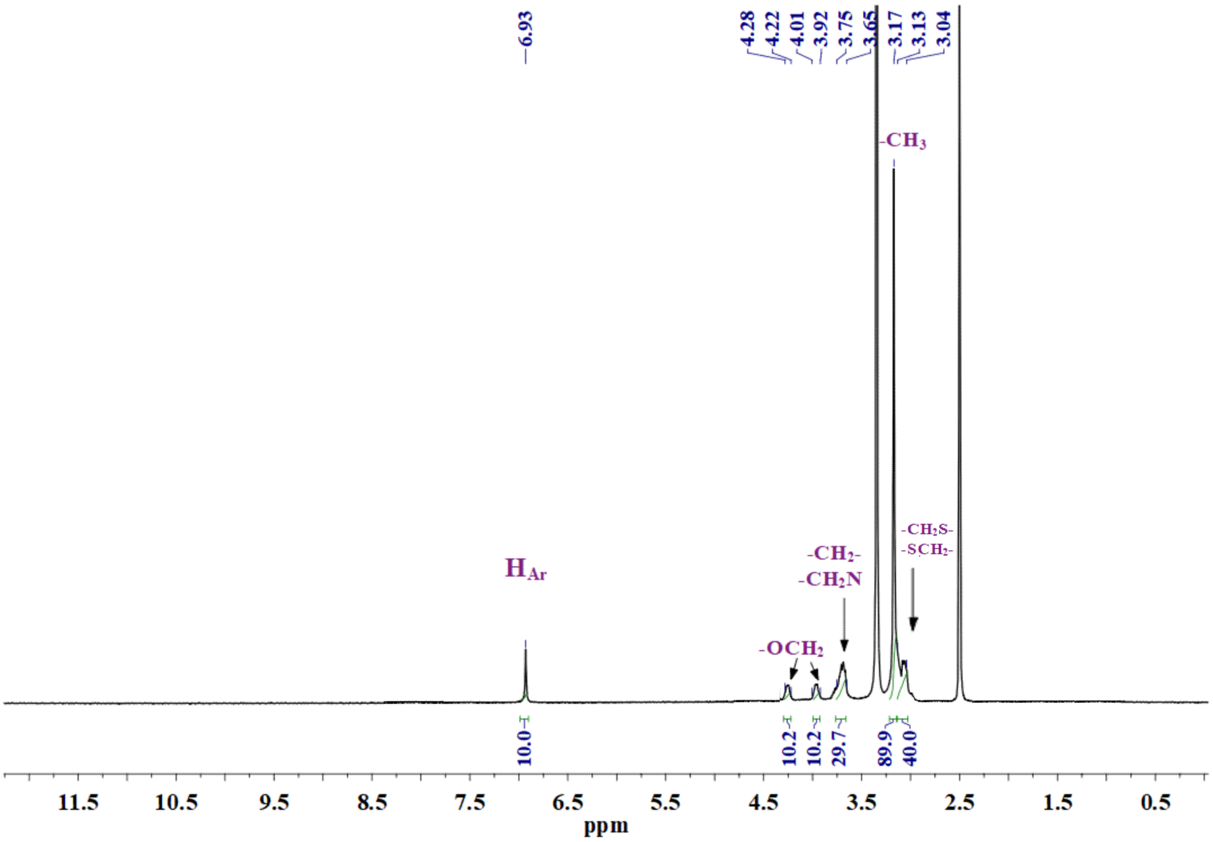

Figure S3. ^{1}H NMR spectrum of 4,8,14,18,23,26,28,31,32,35-deca-[thioethane(2'-(N,N-dimethyl, N-ethyl)ammonium)ethoxy]-pillar[5]arene iodide **(4)**. DMSO-d_6, 298 K, 400 MHz.

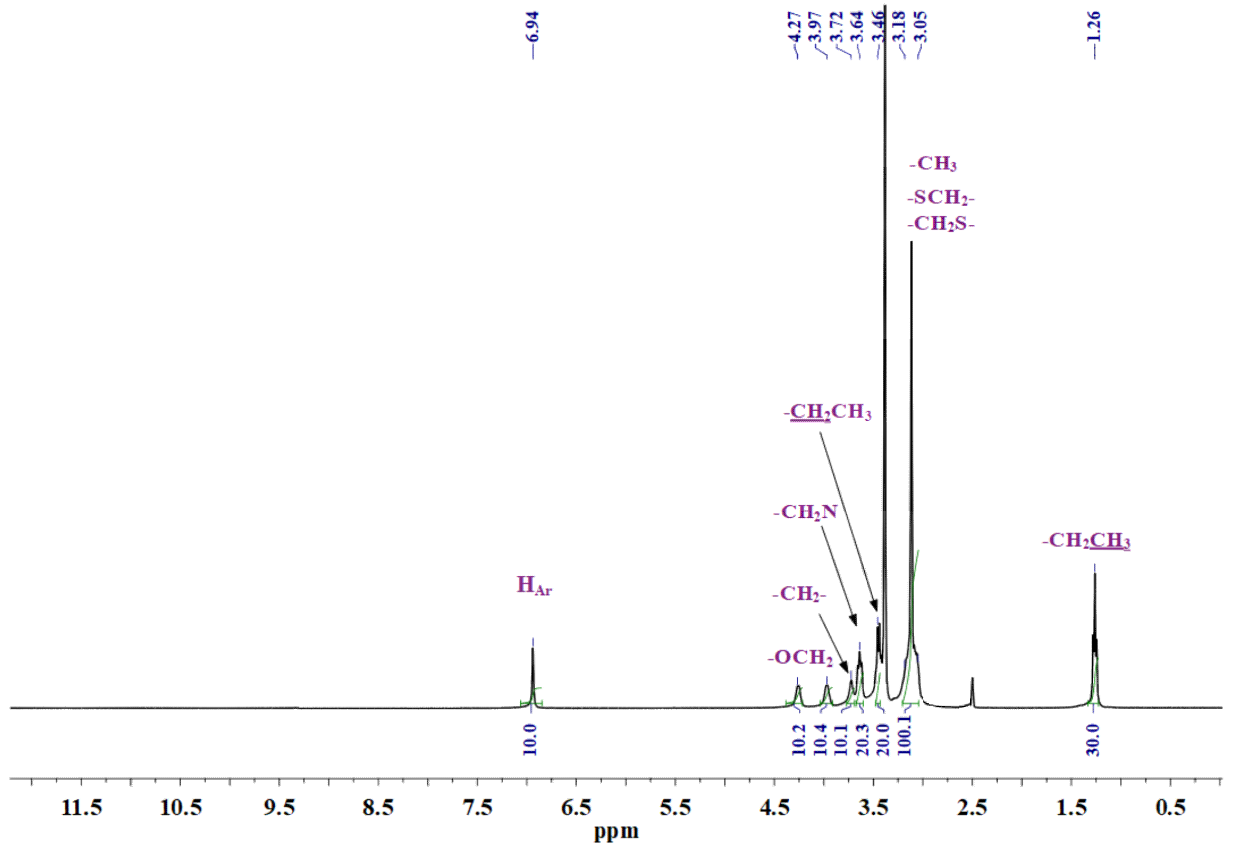

Figure S4. ^{1}H NMR spectrum of 4,8,14,18,23,26,28,31,32,35-deca-[thioethane(2-(N,N-dimethyl, N-acetylethoxy)ammonium)ethoxy]-pillar[5]arene bromide **(5)**. DMSO-d_6, 298 K, 400 MHz.

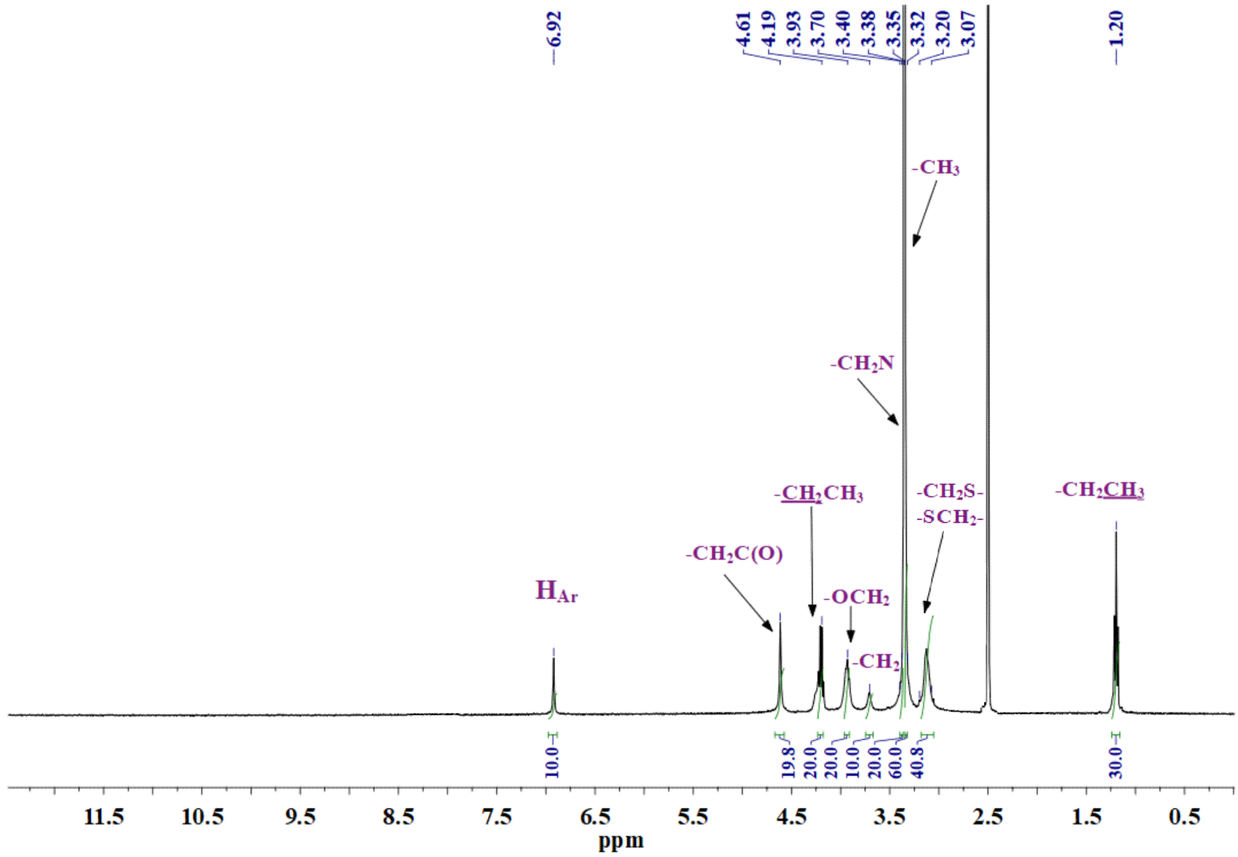

Figure S5. ^{1}H NMR spectrum of 4,8,14,18,23,26,28,31,32,35-deca-[thioethane(2'-(N,N-dimethyl, N-methylcarbamate(N-4'-benzylsulfamide))ammonium)ethoxy]-pillar[5]arene bromide **(6)**. DMSO-d_6, 298 K, 400 MHz.

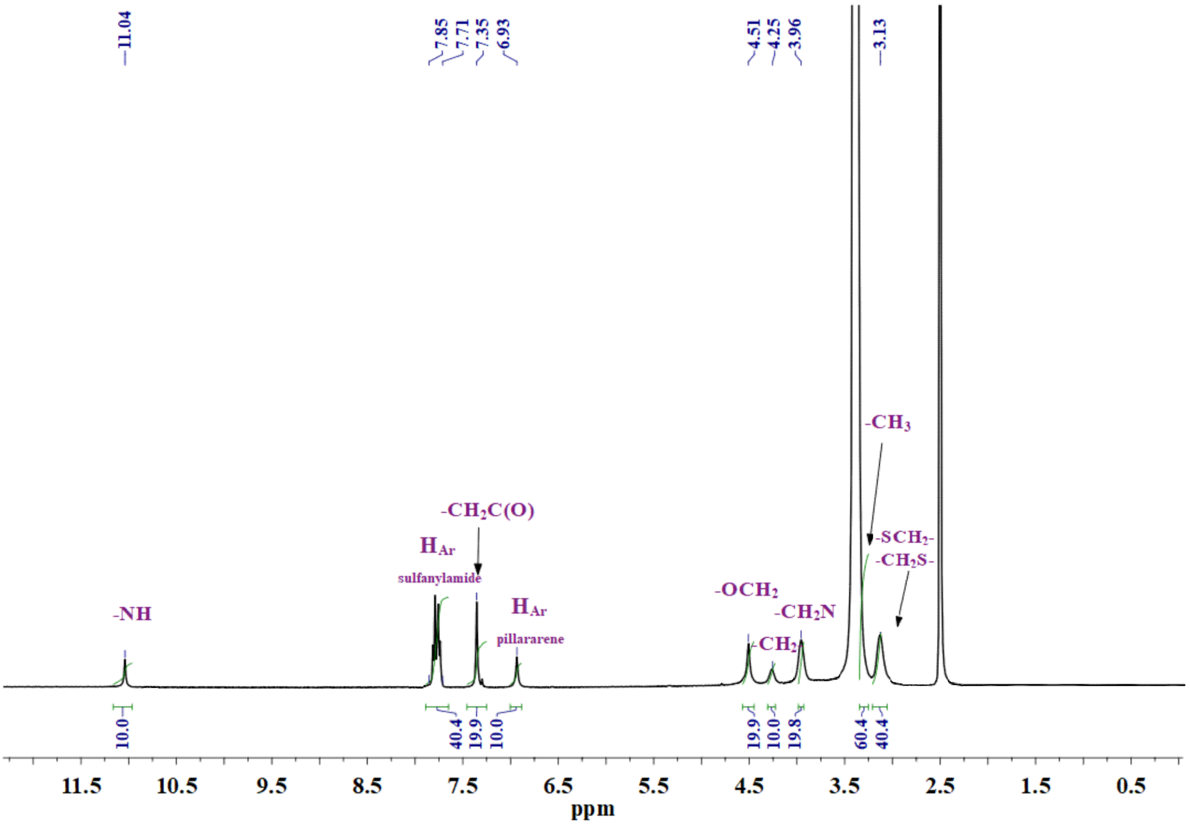

Figure S6. ^{1}H NMR spectrum of 4,8,14,18,23,26,28,31,32,35-deca-[thioethane(2'-(N,N - dimethyl, N-benzyl)ammonium)ethoxy]-pillar[5]arene bromide **(7)**. DMSO-d_6, 298 K, 400 MHz.

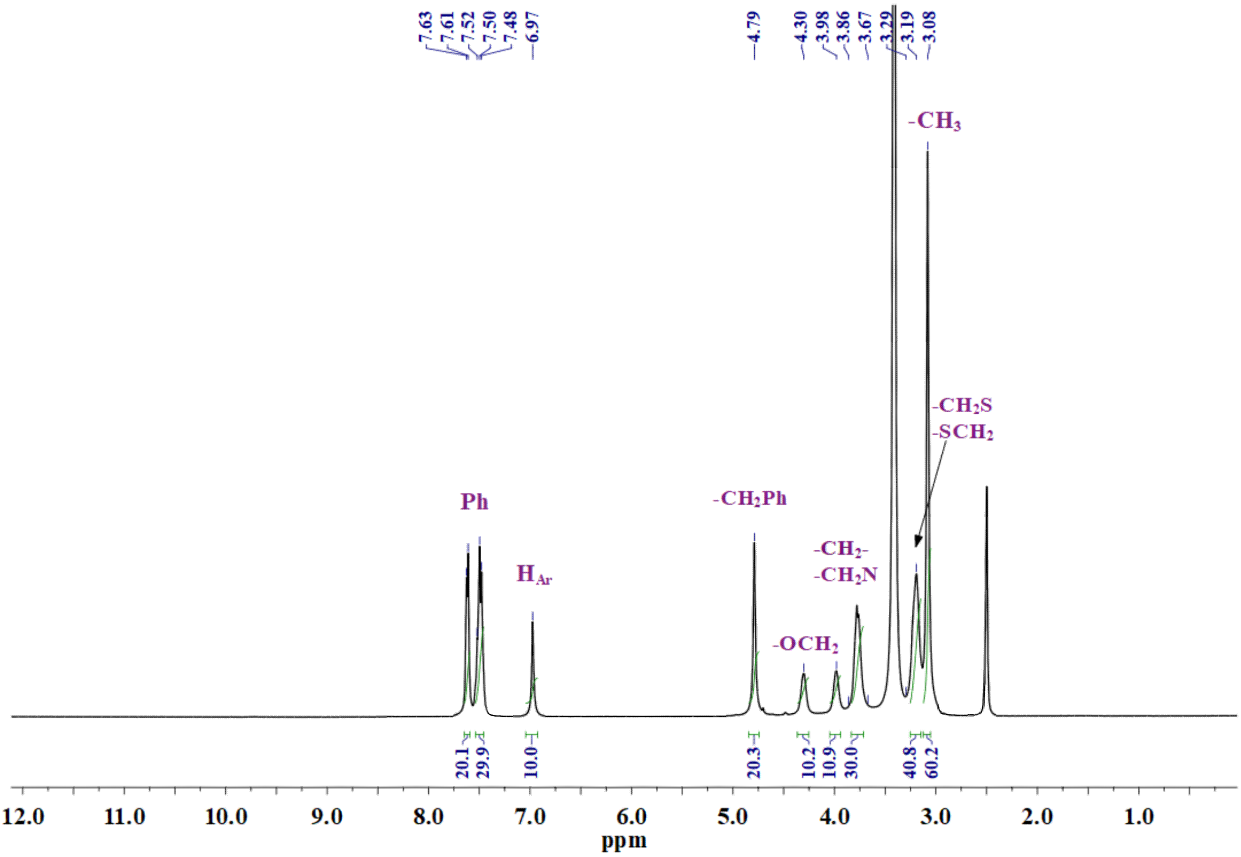

Figure S7. ^{1}H NMR spectrum of 4,8,14,18,23,26,28,31,32,35-deca-[thioethane (2'-(N,N - dimethyl, N-propane-1-sulfonate)ammonium)ethoxy]-pillar[5]arene **(8)**. D_2O, 298 K, 400 MHz.

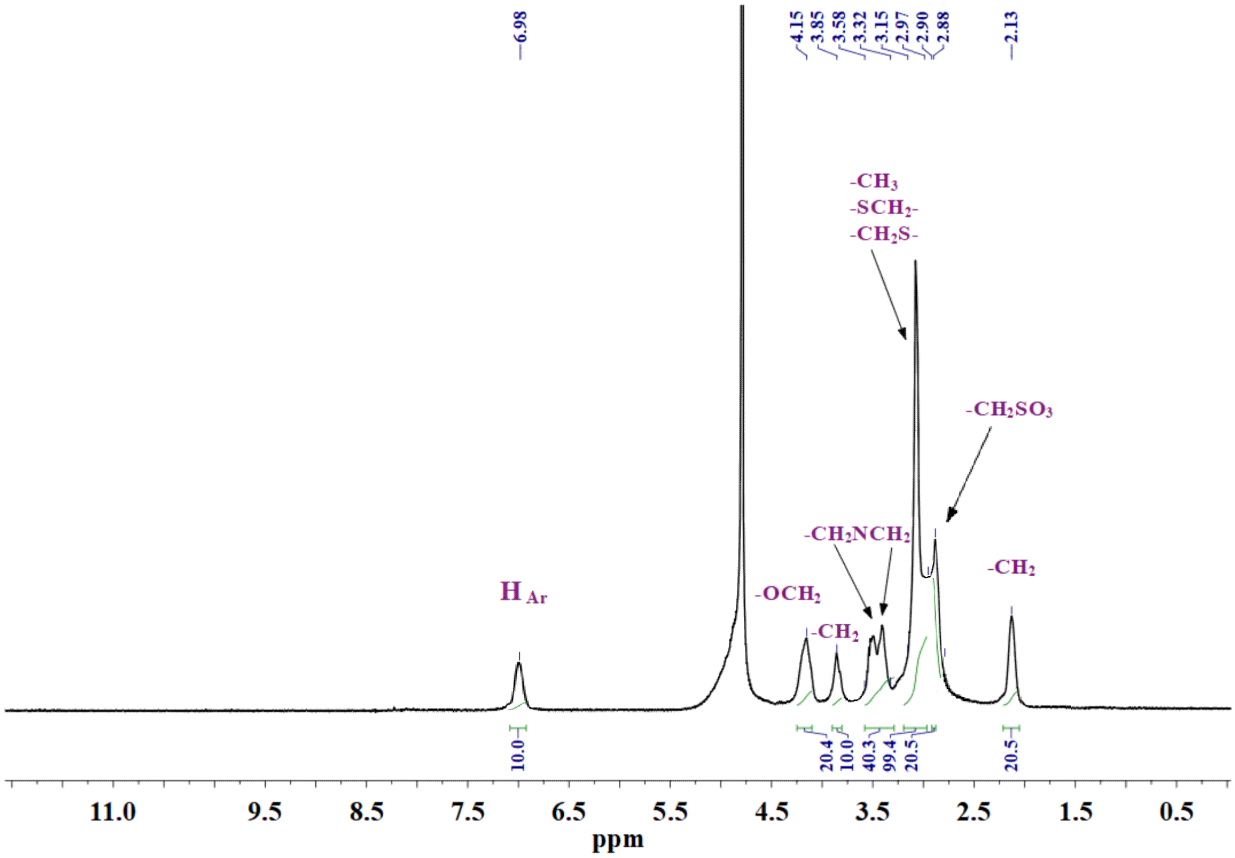

Figure S8. ^{1}H NMR spectrum of 4,8,14,18,23,26,28,31,32,35-deca-[thioethane(2'-(N,N - dimethyl, N-acetate)ammonium)ethoxy]-pillar[5]arene **(9)**. DMSO-d_6, 298 K, 400 MHz.

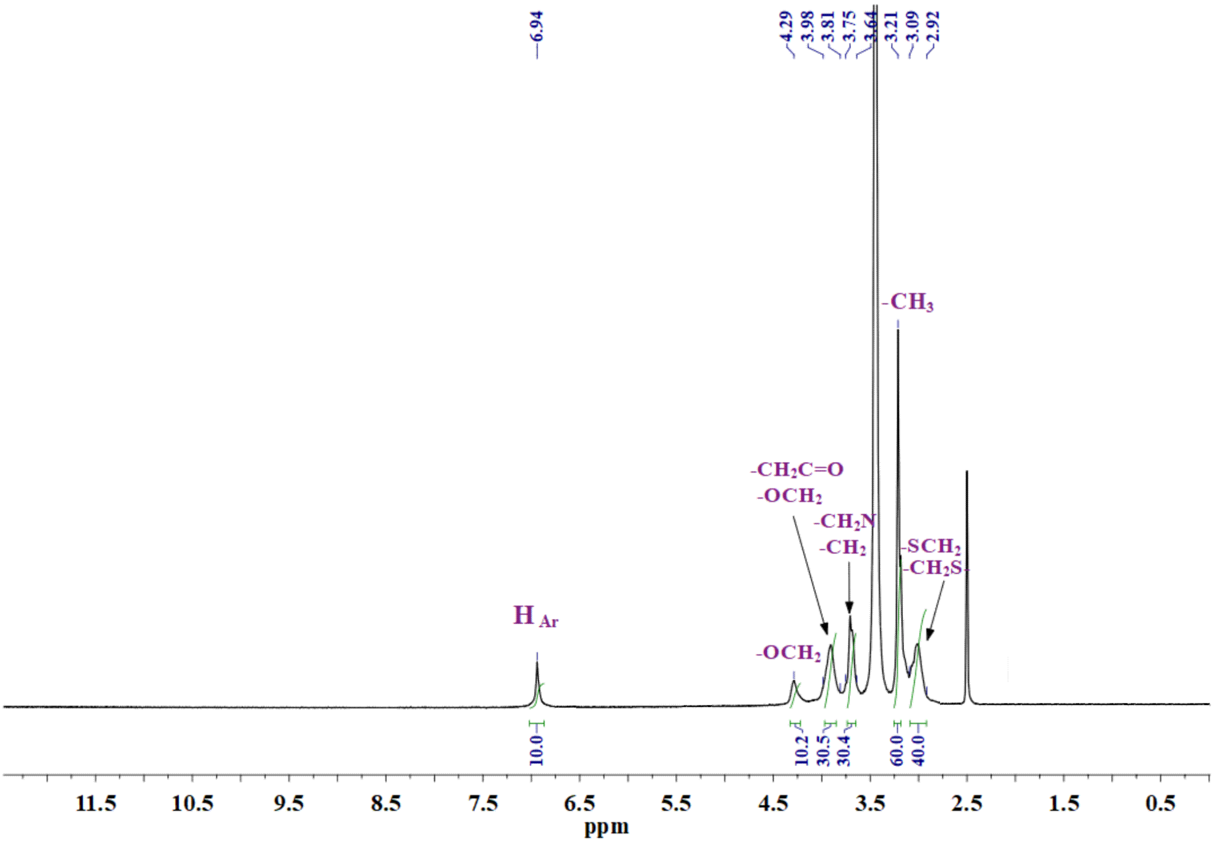

Figure S9. ^{13}C NMR spectrum of 4,8,14,18,23,26,28,31,32,35-deca-[thioethane(2'-(N,N-dimethyl)amino)ethoxy]-pillar[5]arene **(2)**. $CDCl_3$, 298 K, 100 MHz.

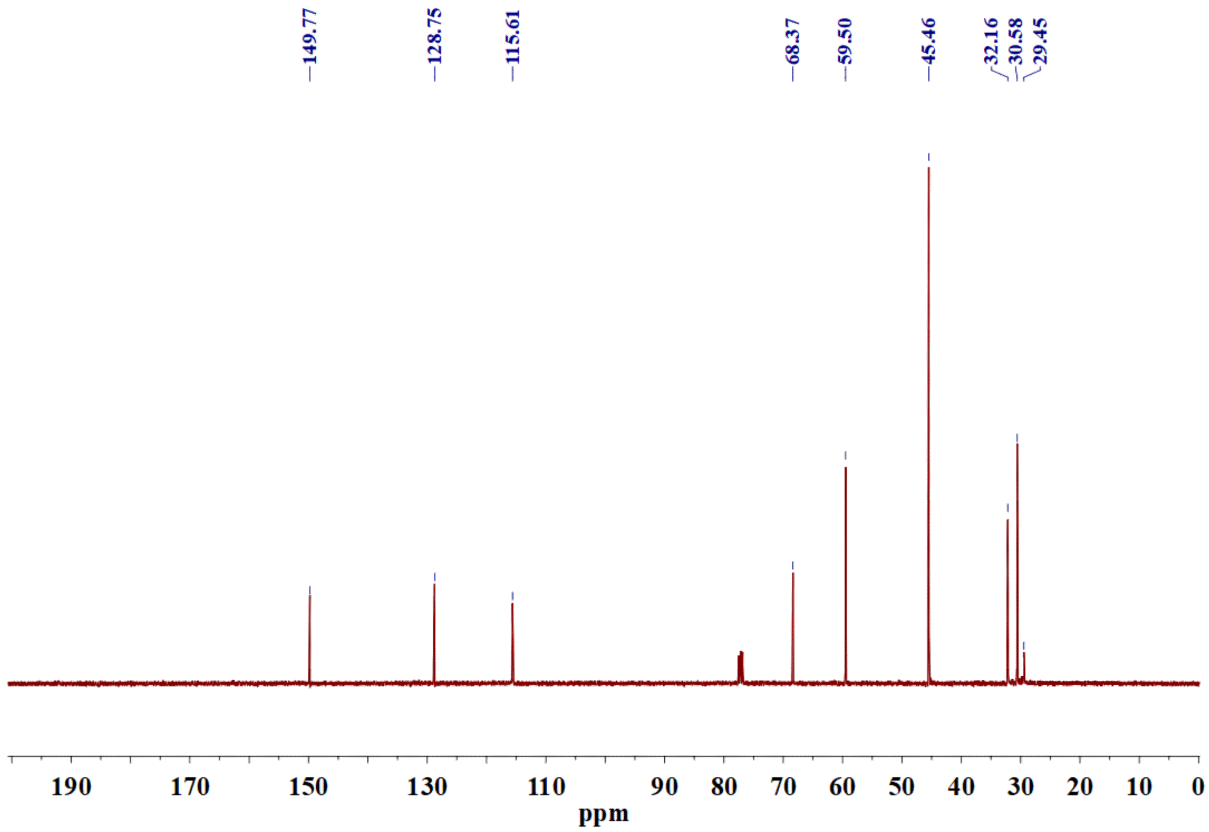

Figure S10. ^{13}C NMR spectrum of 4,8,14,18,23,26,28,31,32,35-deca-[thioethane(2'-(N,N,N-trimethyl)ammonium)ethoxy]-pillar[5]arene iodide **(3)**. DMSO-d_6, 298 K, 100 MHz.

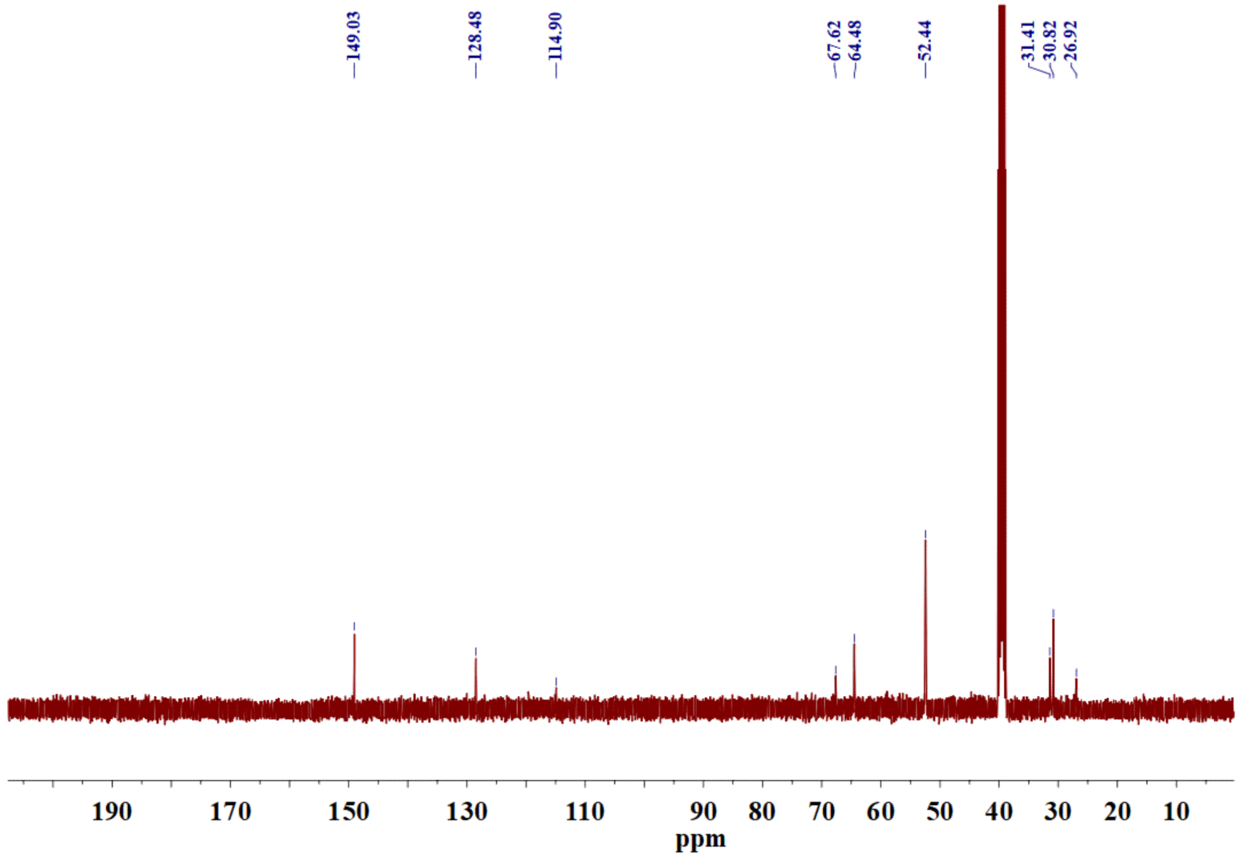

Figure S11. ^{13}C NMR spectrum of 4,8,14,18,23,26,28,31,32,35-deca-[thioethane(2'-(N,N-dimethyl, N-ethyl)ammonium)ethoxy]-pillar[5]arene iodide **(4)**. DMSO-d_6, 298 K, 100 MHz.

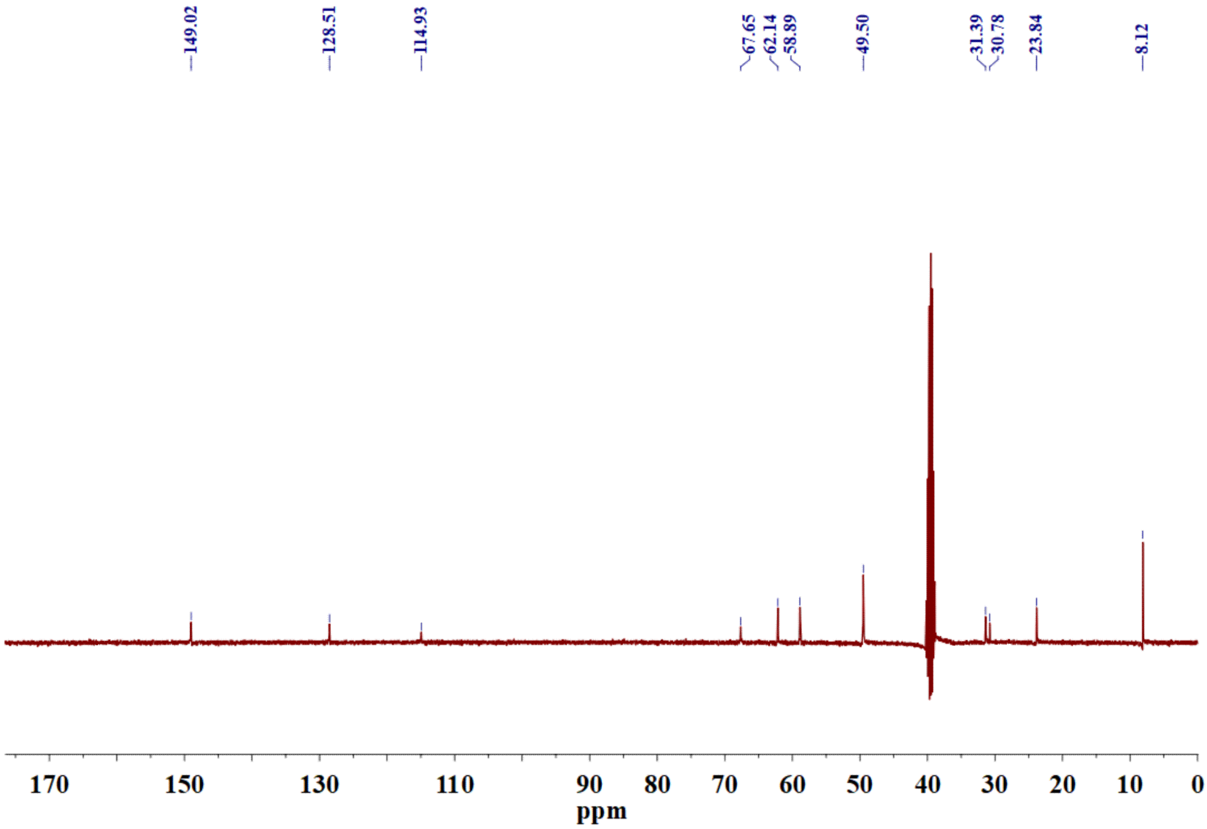

Figure S12. ^{13}C NMR spectrum of 4,8,14,18,23,26,28,31,32,35-deca-[thioethane(2-(N,N-dimethyl, N-acetylethoxy)ammonium)ethoxy]-pillar[5]arene bromide **(5)**. DMSO-d_6, 298 K, 100 MHz.

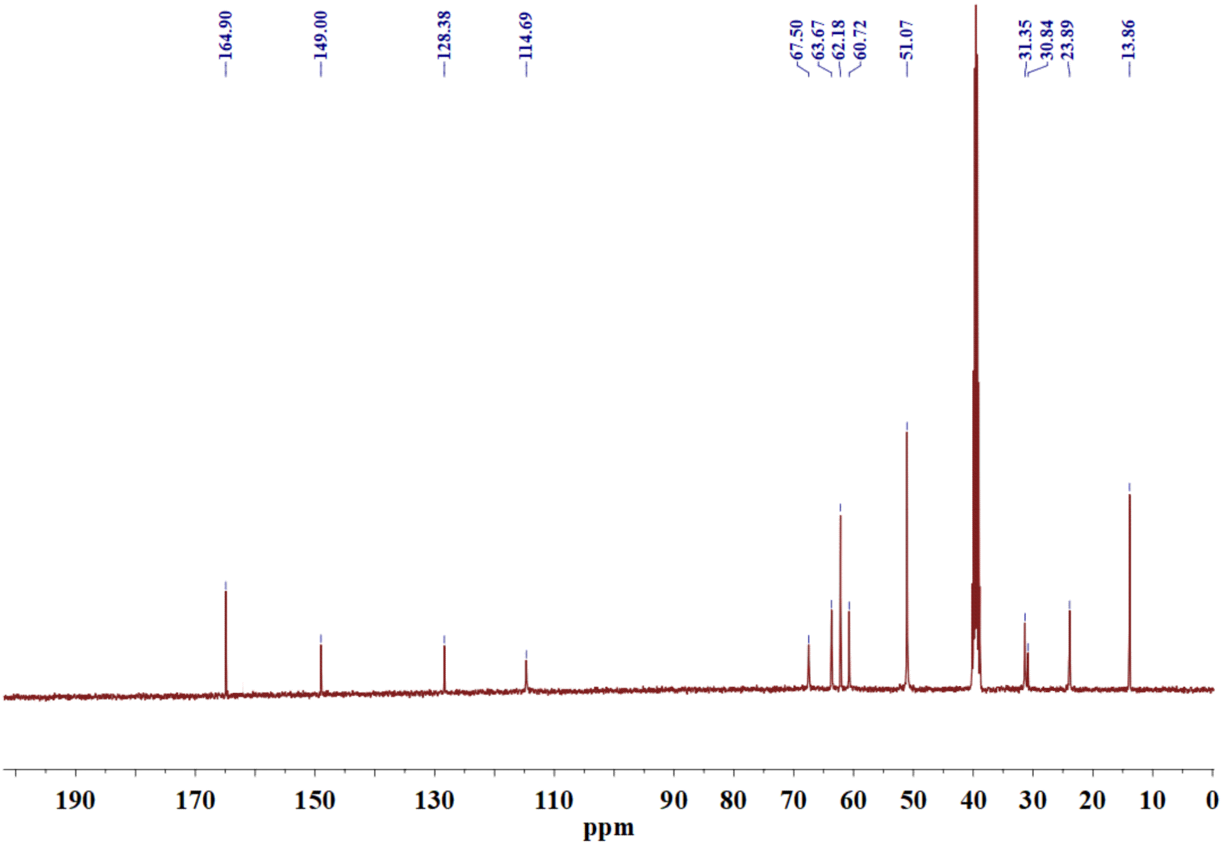

Figure S13. ^{13}C NMR spectrum of 4,8,14,18,23,26,28,31,32,35-deca-[thioethane(2'-(N,N-dimethyl, N-methylcarbamate(N-4'-benzylsulfamide))ammonium)ethoxy]-pillar[5]arene bromide **(6)**. DMSO-d_6, 298 K, 100 MHz.

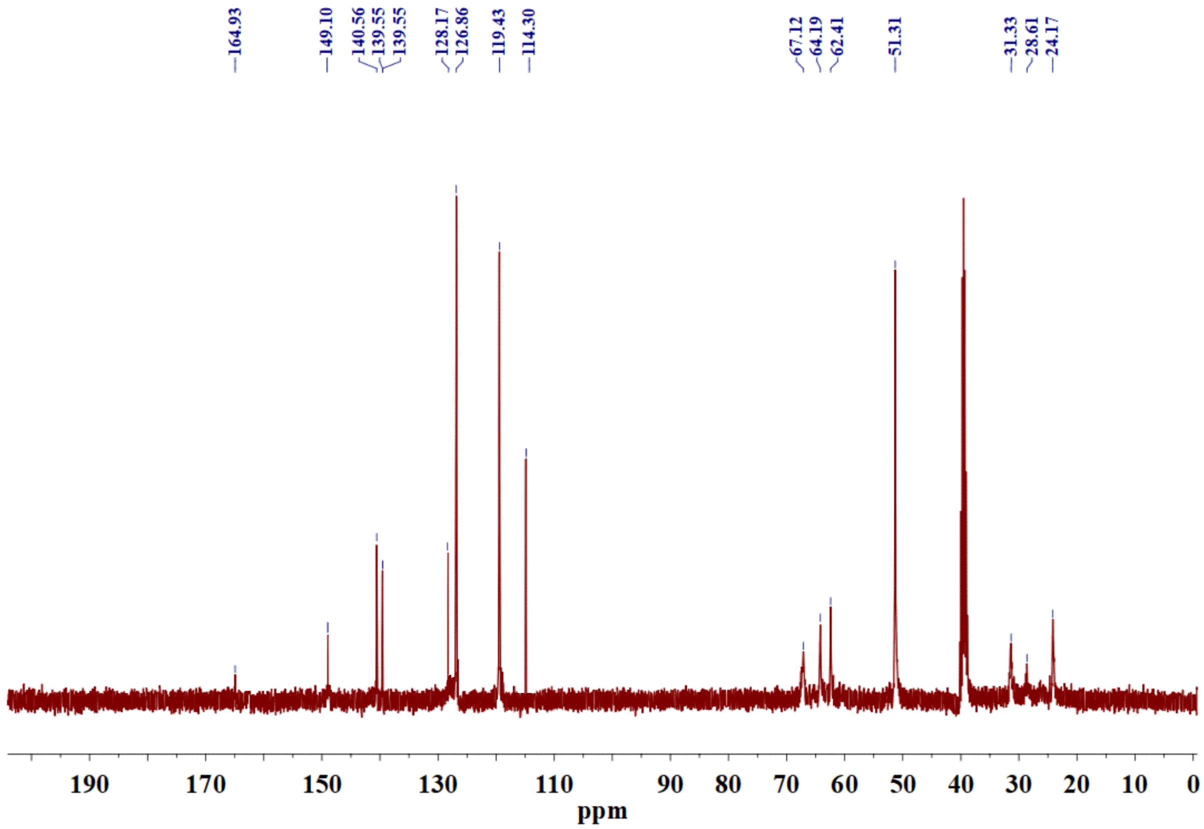

Figure S14. ^{13}C NMR spectrum of 4,8,14,18,23,26,28,31,32,35-deca-[thioethane(2'-(N,N - dimethyl, N-benzyl)ammonium)ethoxy]-pillar[5]arene bromide **(7)**. DMSO-d_6, 298 K, 100 MHz.

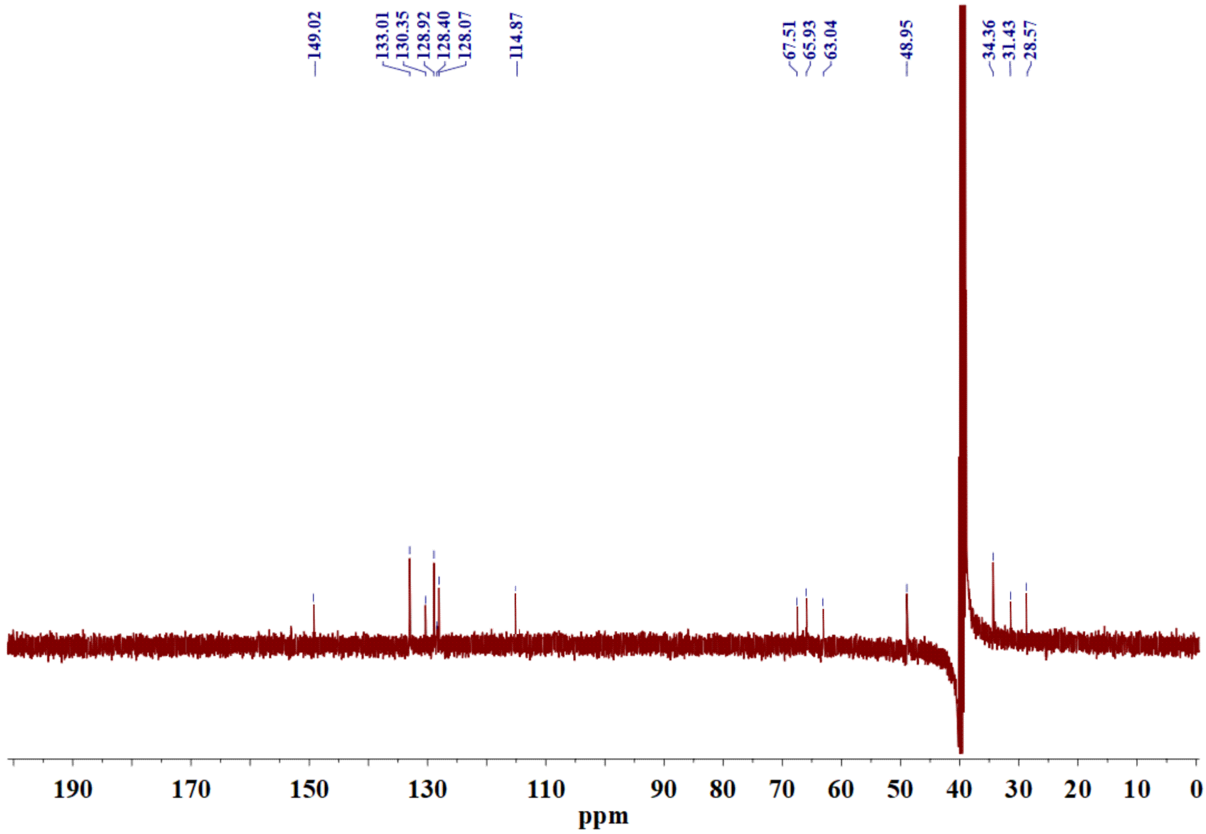

Figure S15. ^{13}C NMR spectrum of 4,8,14,18,23,26,28,31,32,35-deca-[thioethane(2'-(N,N - dimethyl, N-propane-1-sulfonate)ammonium)ethoxy]-pillar[5]arene **(8)**. DMSO-d_6, 298 K, 100 MHz.

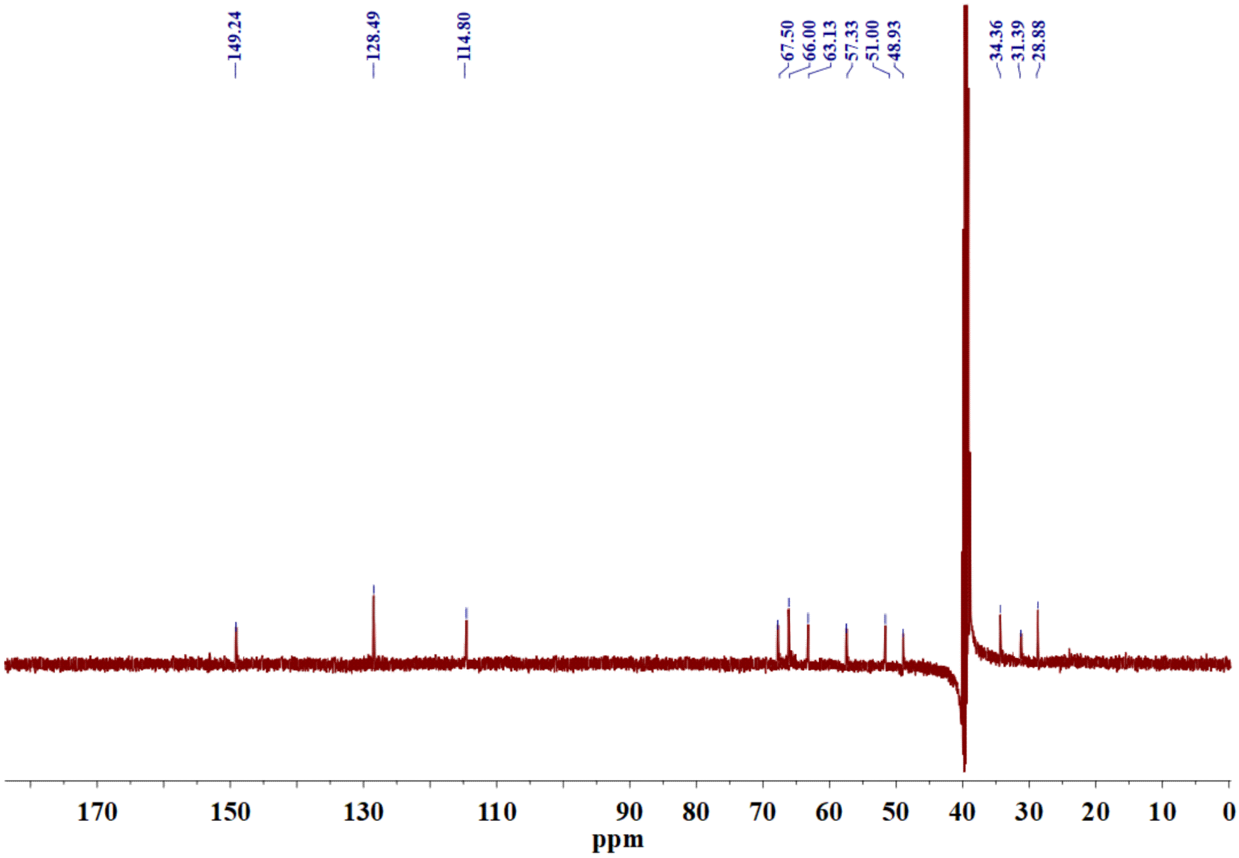

Figure S16. ^{13}C NMR spectrum of 4,8,14,18,23,26,28,31,32,35-deca-[thioethane (2'-(N,N - dimethyl, N-acetate)ammonium)ethoxy]-pillar[5]arene **(9)**. DMSO-d_6, 298 K, 100 MHz.

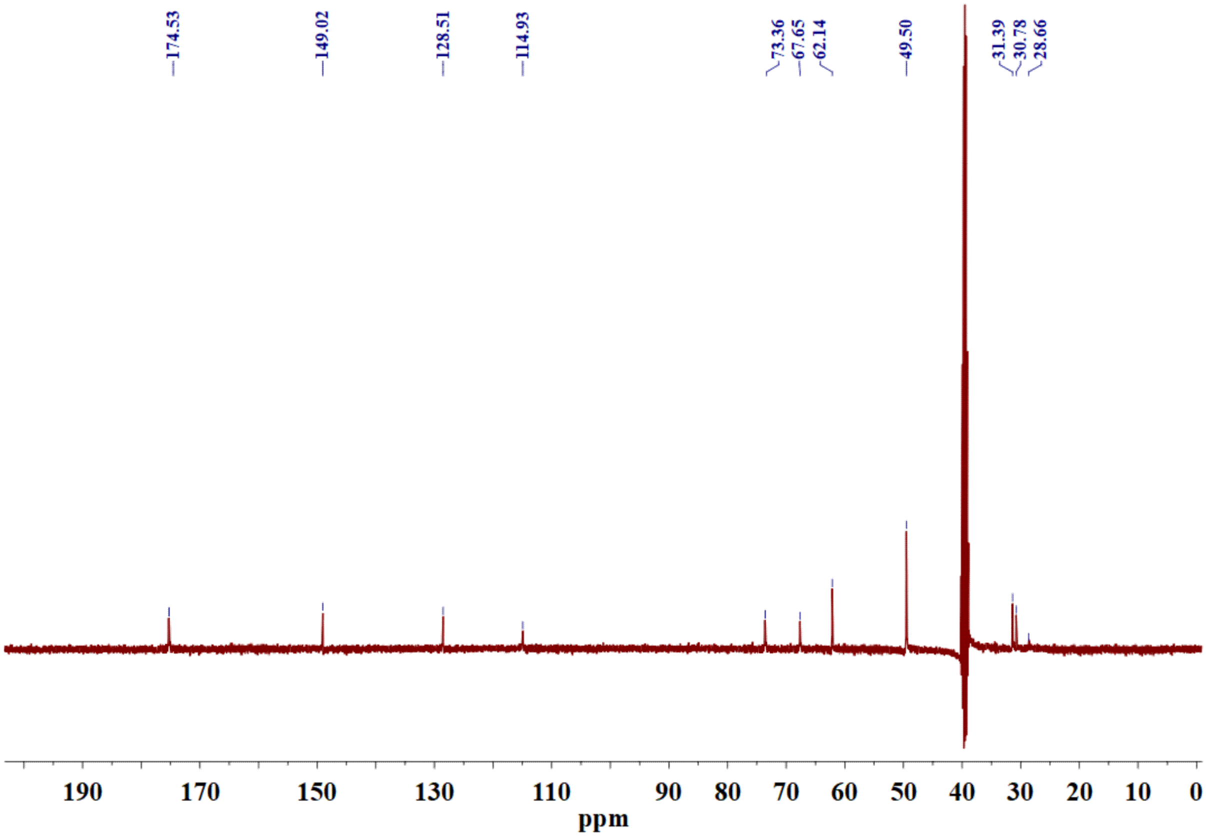

Figure S17. Mass spectrum (MALDI-TOF, 4-nitroaniline matrix) of 4,8,14,18,23,26,28,31,32,35-deca-[thioethane(2'-(N,N-dimethyl)amino)ethoxy]-pillar[5]arene **(2)**.

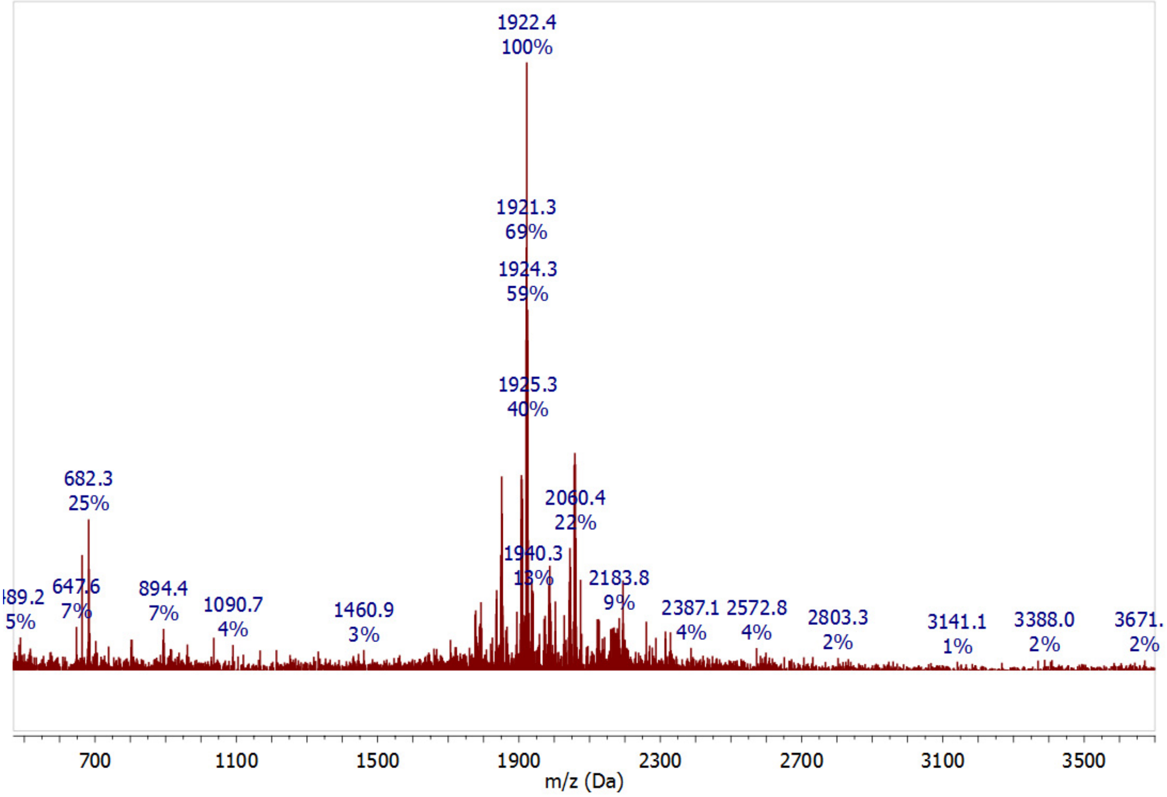

Figure S18. Mass spectrum (ESI) of 4,8,14,18,23,26,28,31,32,35-deca-[thioethane(2'-(N,N,N-trimethyl)ammonium)ethoxy]-pillar[5]arene iodide **(3)**.

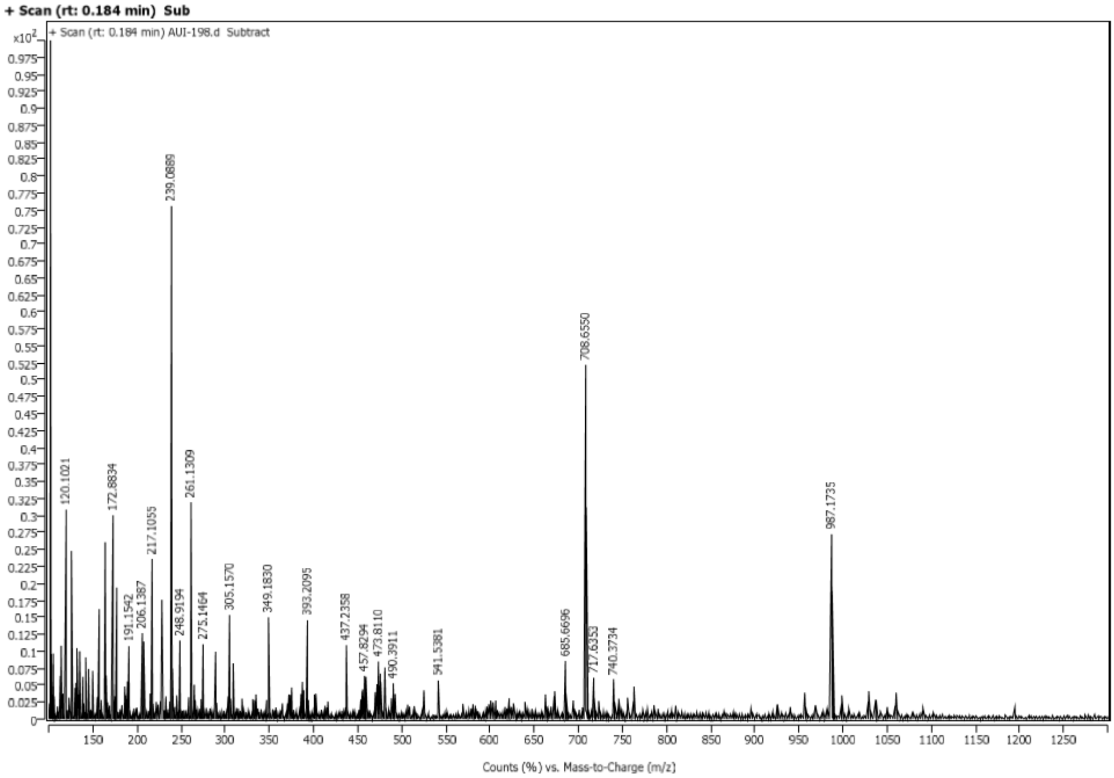

Figure S19. Mass spectrum (ESI) of 4,8,14,18,23,26,28,31,32,35-deca-[thioethane(2'-(N,N-dimethyl, N-ethyl)ammonium)ethoxy]-pillar[5]arene iodide **(4)**.

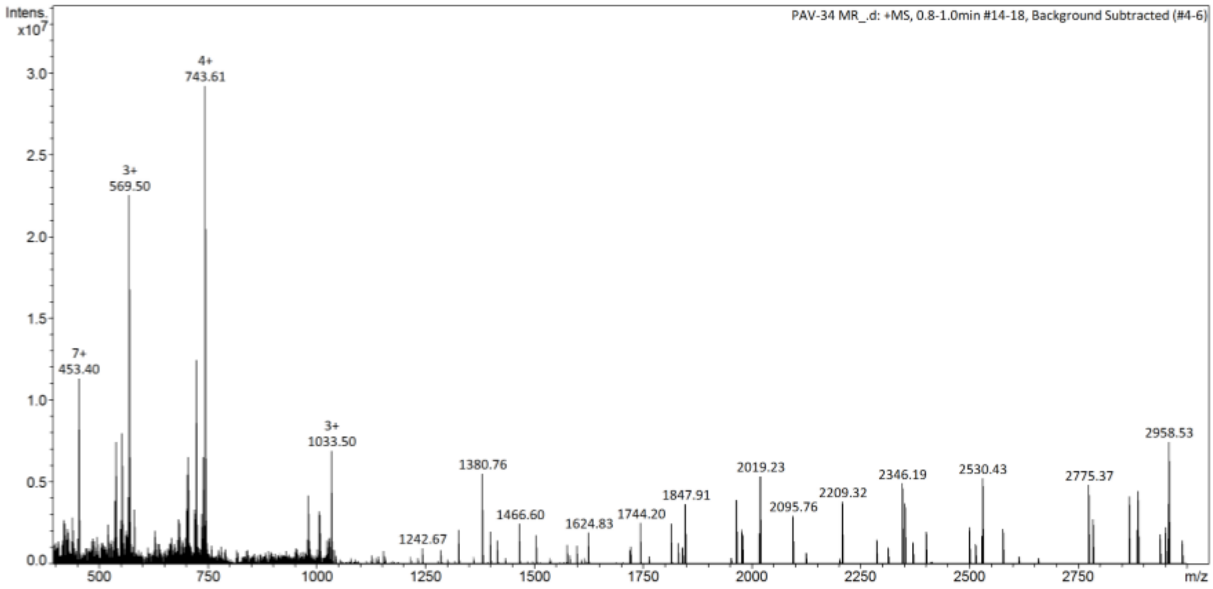

Figure S20. Mass spectrum (ESI) of 4,8,14,18,23,26,28,31,32,35-deca-[thioethane(2-(N,N-dimethyl, N-acetylethoxy)ammonium)ethoxy]-pillar[5]arene bromide **(5)**.

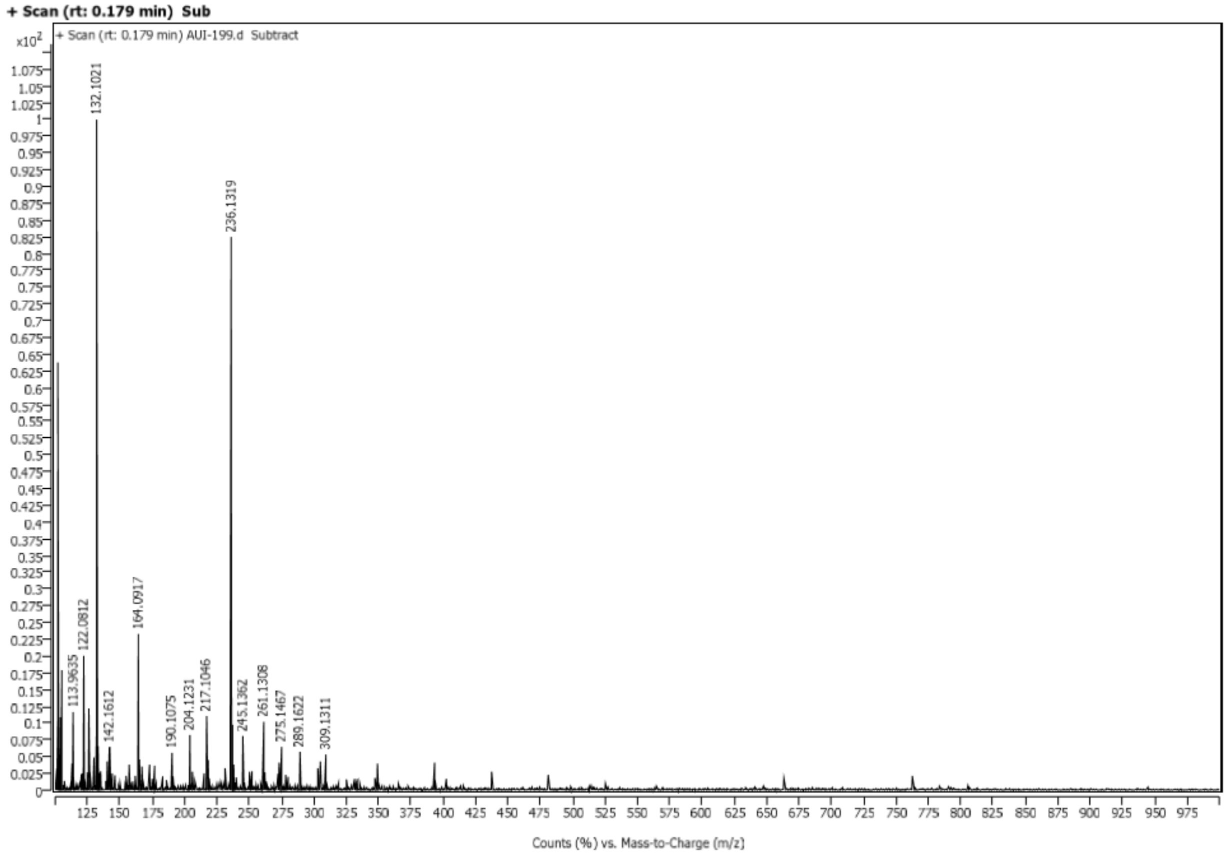

Figure S21. Mass spectrum (ESI) of 4,8,14,18,23,26,28,31,32,35-deca-[thioethane(2'-(N,N-dimethyl, N-methylcarbamate(N-4'-benzylsulfamide))ammonium)ethoxy]-pillar[5]arene bromide **(6)**.

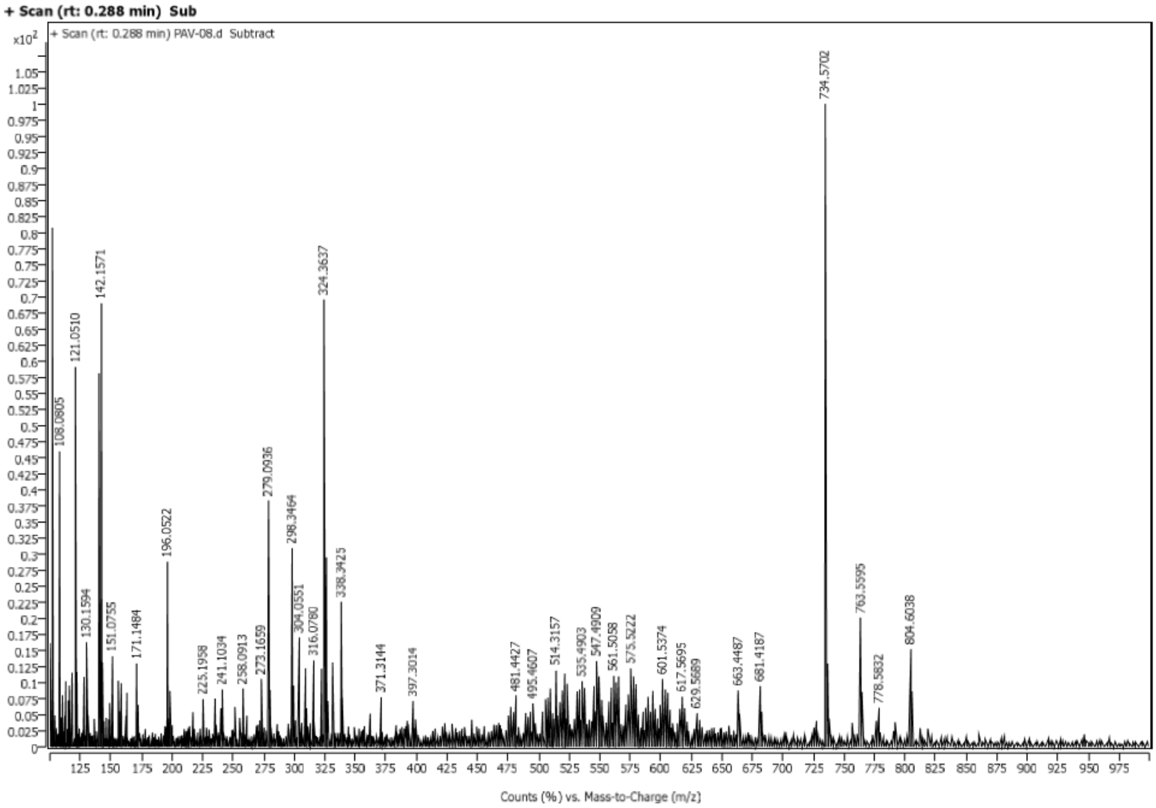

Figure S22. Mass spectrum (ESI), 4-nitroaniline matrix) of 4,8,14,18,23,26,28,31,32,35-deca-[thioethane (2'-(N,N - dimethyl, N-benzyl)ammonium)ethoxy]-pillar[5]arene bromide **(7)**.

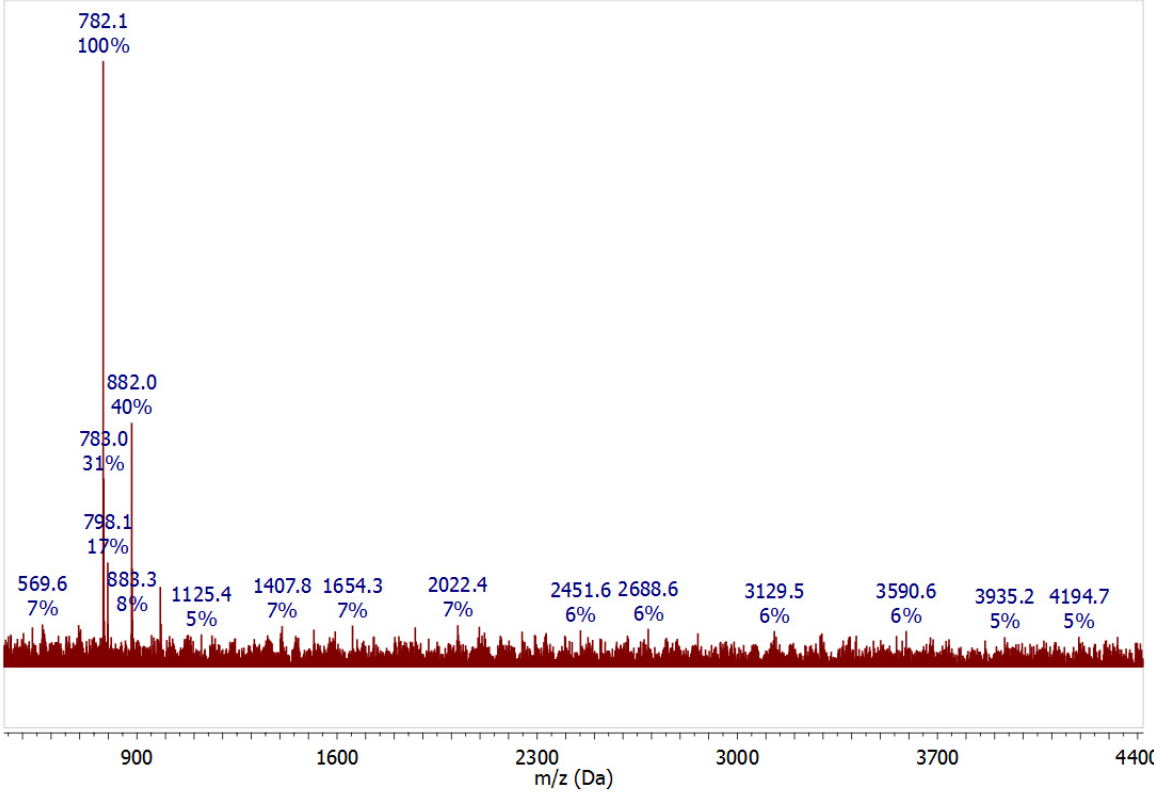

Figure S23. Mass spectrum (ESI) of 4,8,14,18,23,26,28,31,32,35- deca-[thioethane (2'-(N,N - dimethyl, N-propane-1-sulfonate)ammonium)ethoxy]--pillar[5]arene **(8)**.

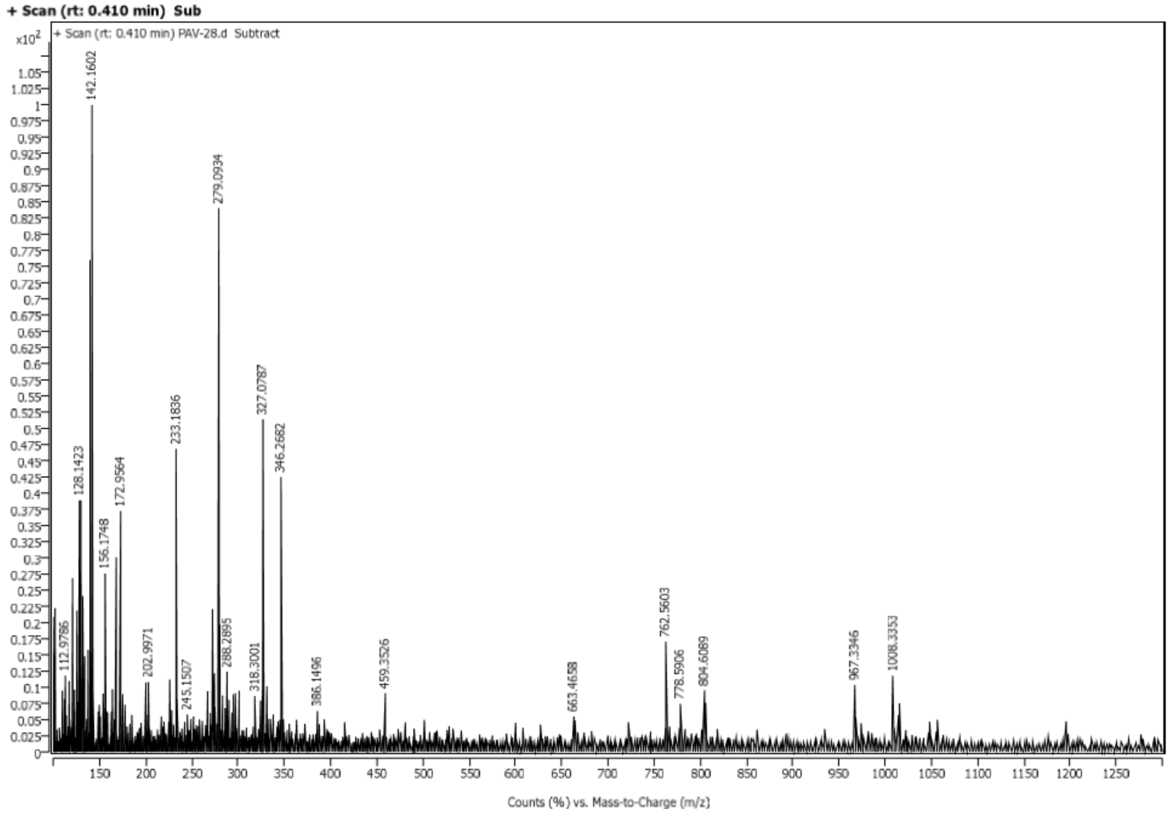

Figure S24. Mass spectrum (ESI) of 4,8,14,18,23,26,28,31,32,35-deca-[thioethane (2'-(N,N - dimethyl, N-acetate)ammonium)ethoxy]-pillar[5]arene **(9)**.

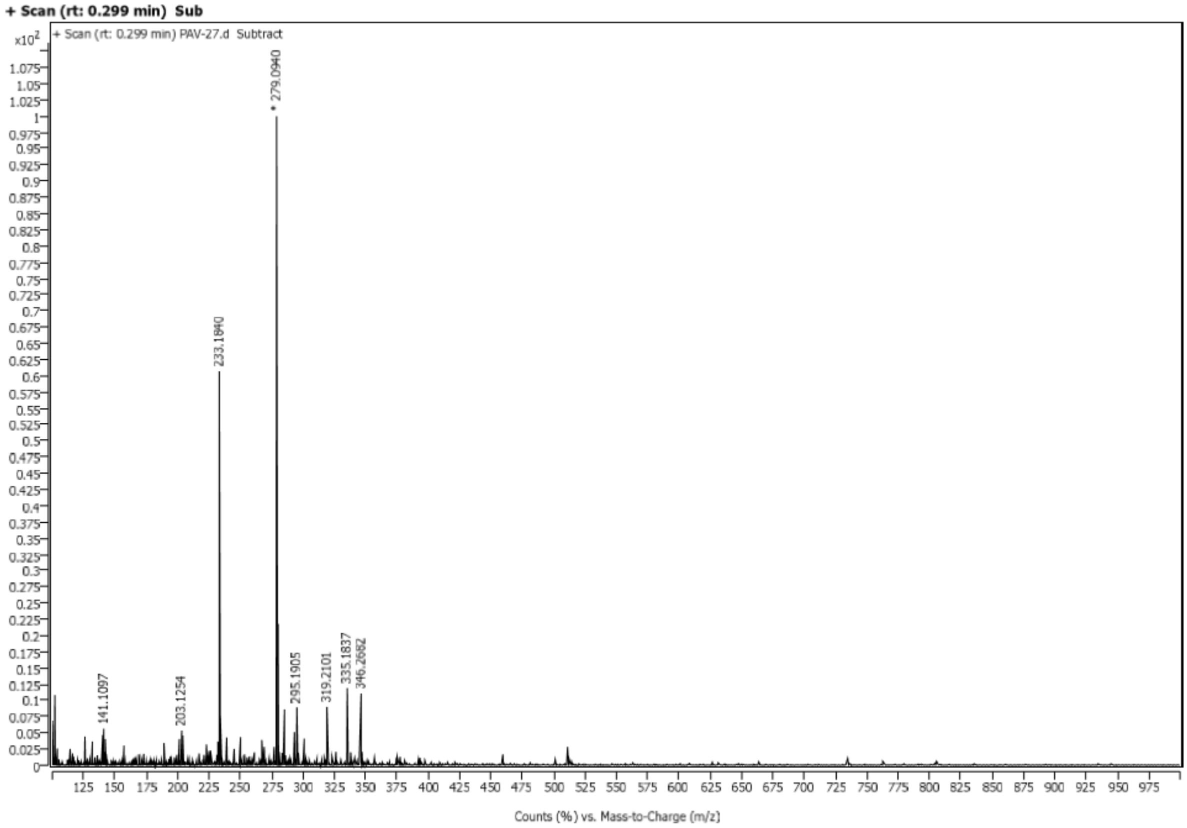

Figure S25. IR spectrum of 4,8,14,18,23,26,28,31,32,35-deca-[thioethane(2'-(N,N-dimethyl)amino)ethoxy]-pillar[5]arene **(2)**.

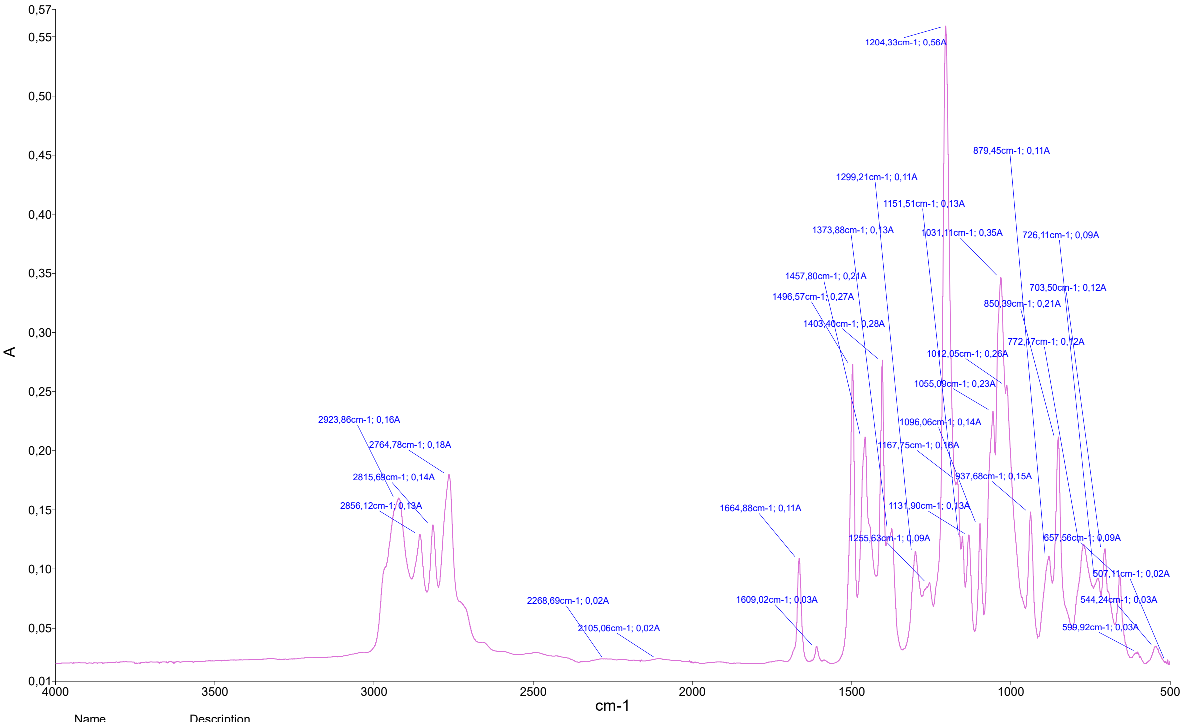

Figure S26. IR spectrum of 4,8,14,18,23,26,28,31,32,35-deca-[thioethane(2'-(N,N,N-trimethyl)ammonium)ethoxy]-pillar[5]arene iodide **(3)**.

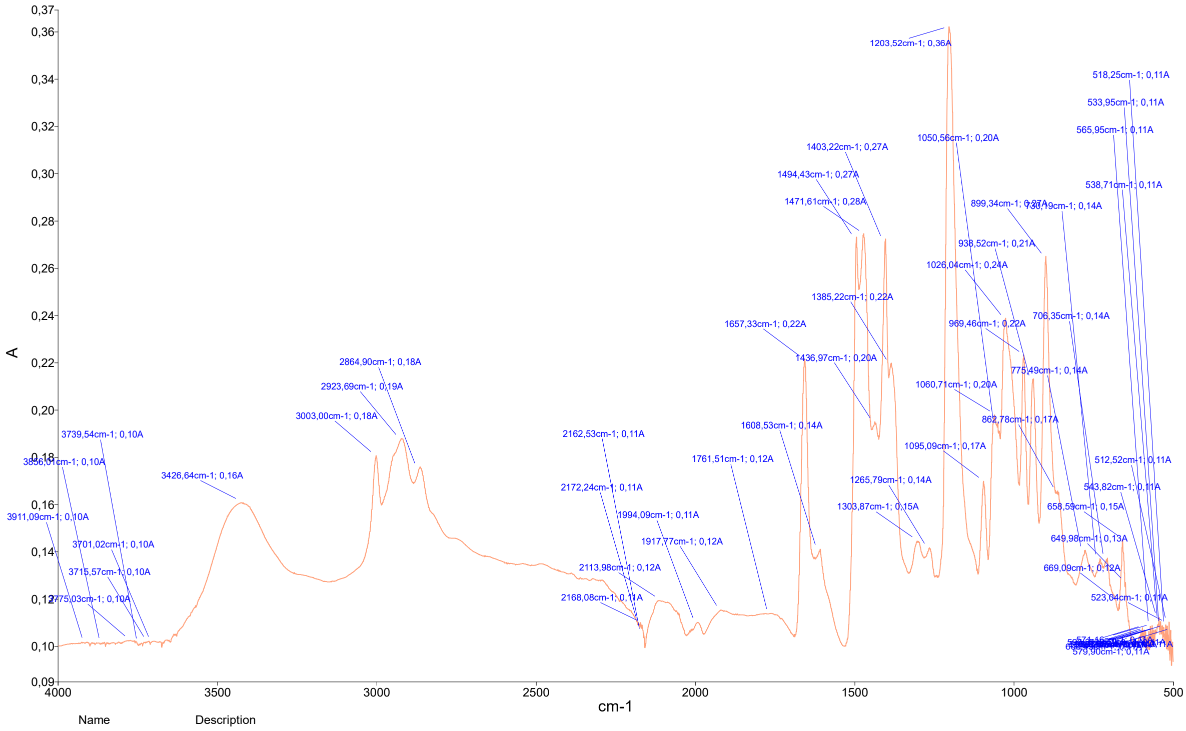

Figure S27. IR spectrum of 4,8,14,18,23,26,28,31,32,35-deca-[thioethane(2'-(N,N-dimethyl, N-ethyl)ammonium)ethoxy]-pillar[5]arene iodide **(4)**.

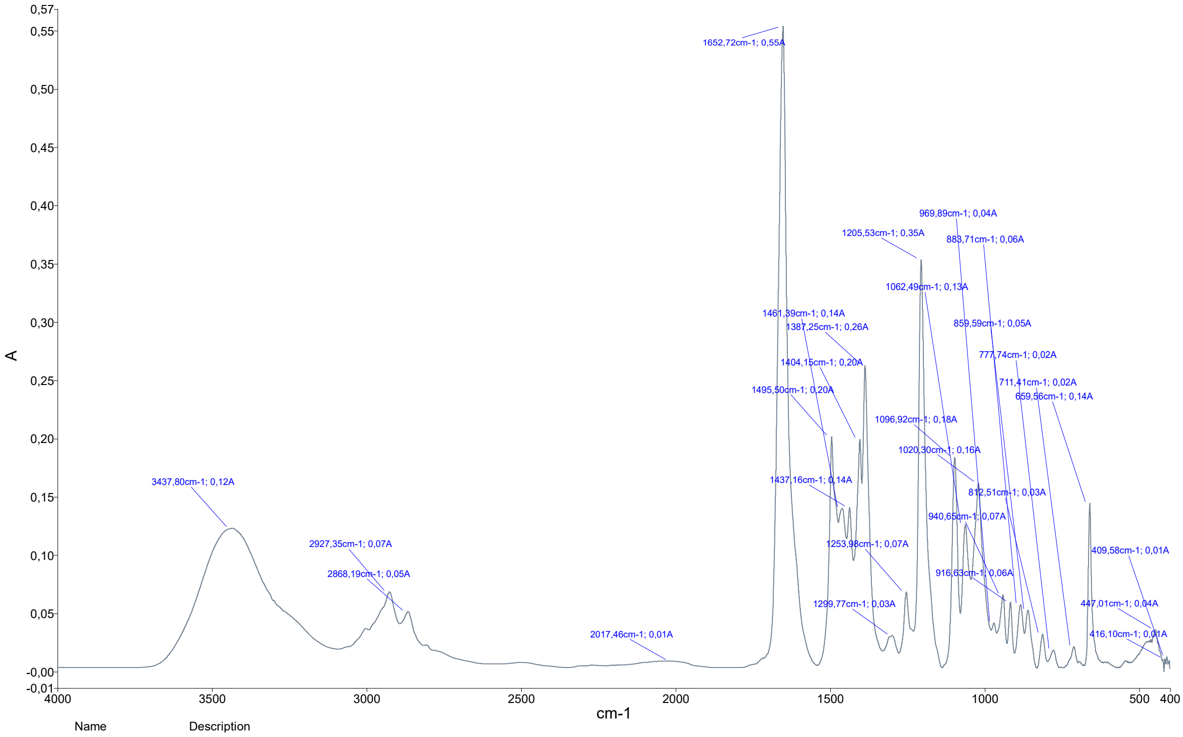

Figure S28. IR spectrum of 4,8,14,18,23,26,28,31,32,35-deca-[thioethane(2-(N,N-dimethyl, N-acetylethoxy)ammonium)ethoxy]-pillar[5]arene bromide **(5)**.

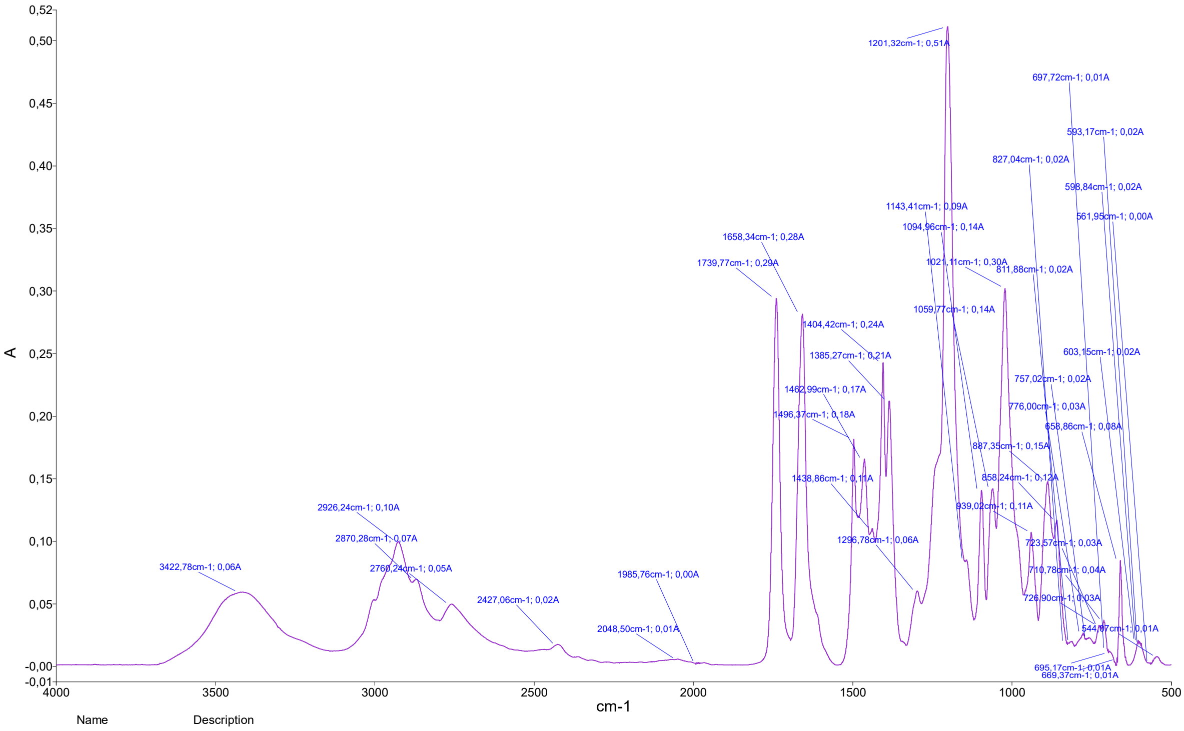

Figure S29. IR spectrum of 4,8,14,18,23,26,28,31,32,35-deca-[thioethane(2'-(N,N-dimethyl, N-methylcarbamate(N-4'-benzylsulfamide))ammonium)ethoxy]-pillar[5]arene bromide **(6)**.

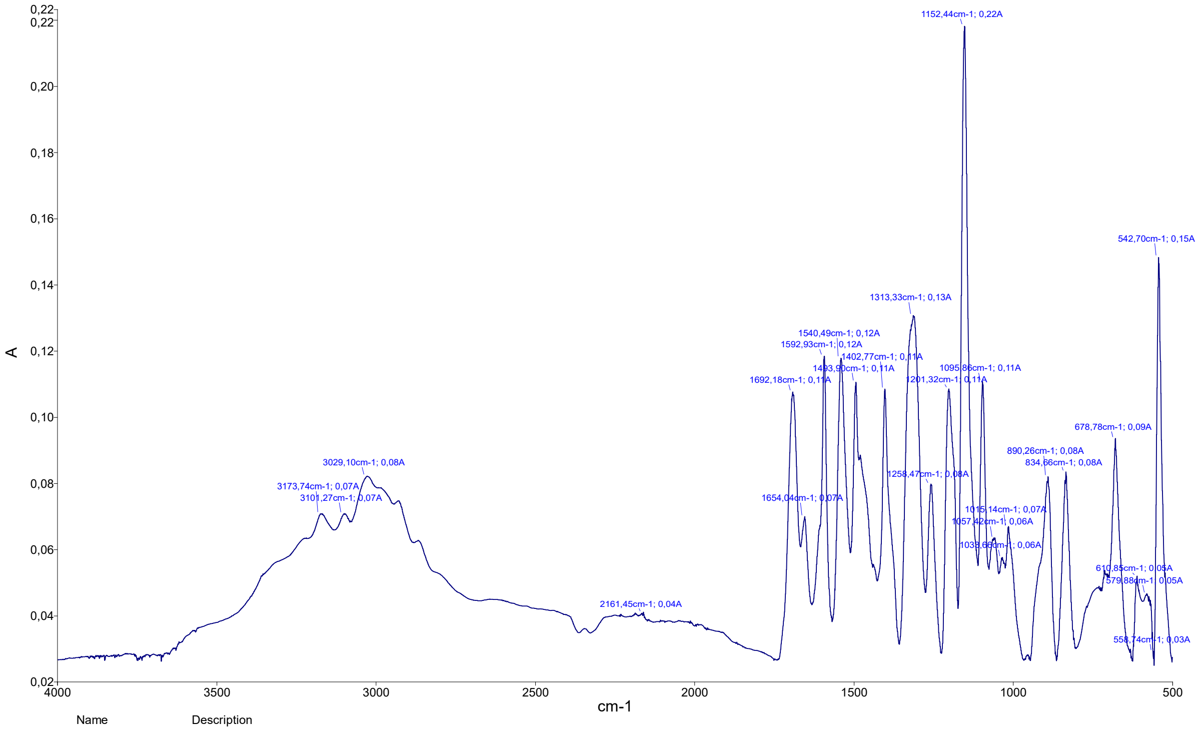

Figure S30. IR spectrum of 4,8,14,18,23,26,28,31,32,35-deca-[thioethane (2'-(N,N - dimethyl, N-benzyl) ammonium)ethoxy]-pillar[5]arene bromide (**7**).

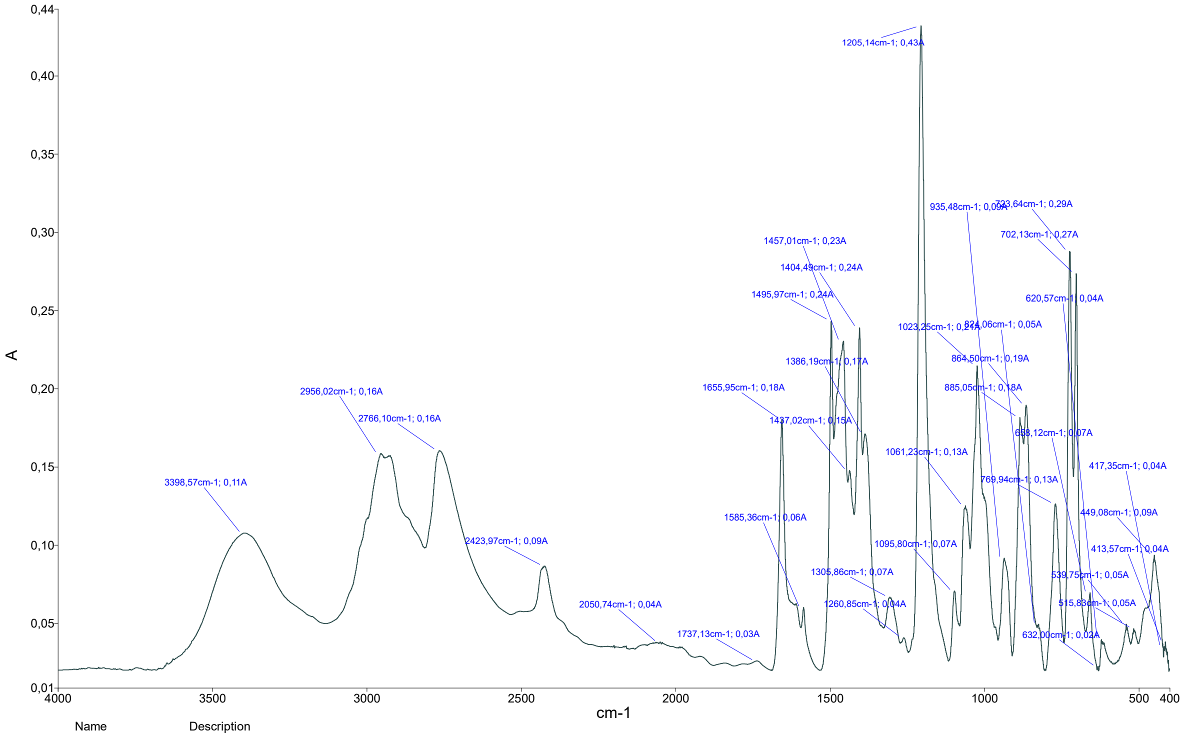

Figure S31. IR spectrum of 4,8,14,18,23,26,28,31,32,35- deca-[thioethane (2'-(N,N - dimethyl, N-propane-1-sulfonate)ammonium)ethoxy]-pillar[5]arene (**8**).

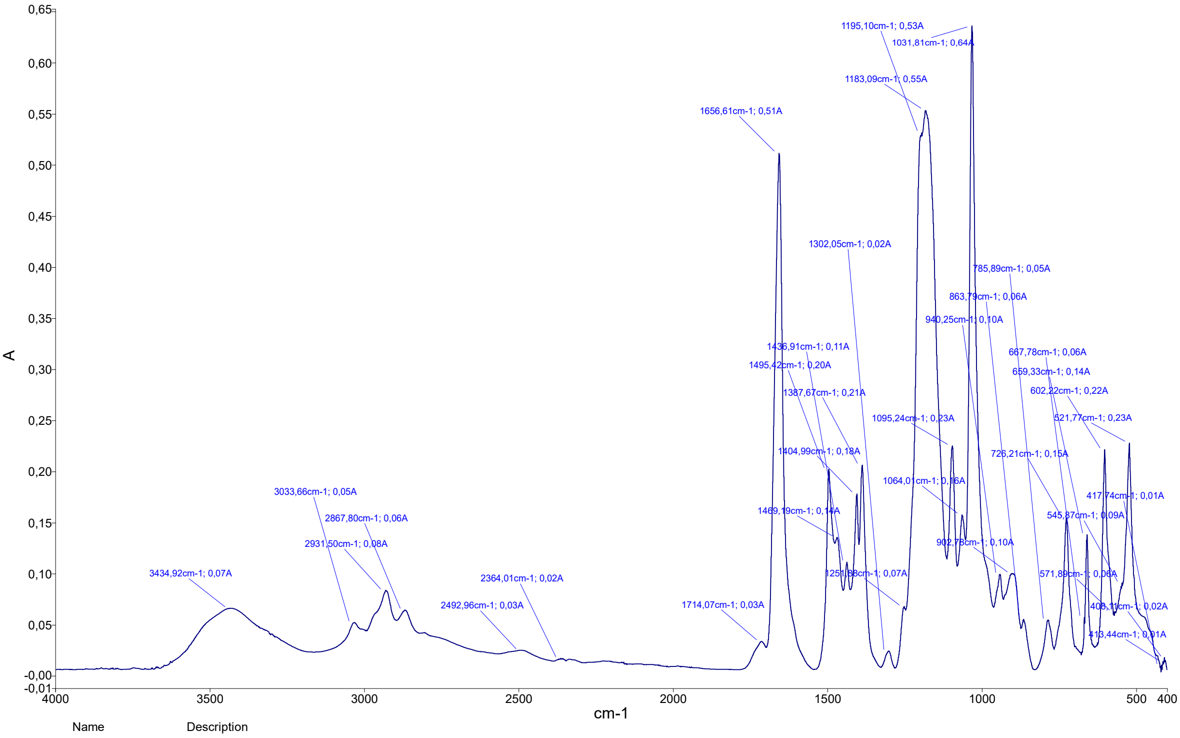

Figure S32. IR spectrum of 4,8,14,18,23,26,28,31,32,35-deca-[thioethane(2'-(N,N - dimethyl, N-acetate)ammonium)ethoxy]-pillar[5]arene **(9)**.

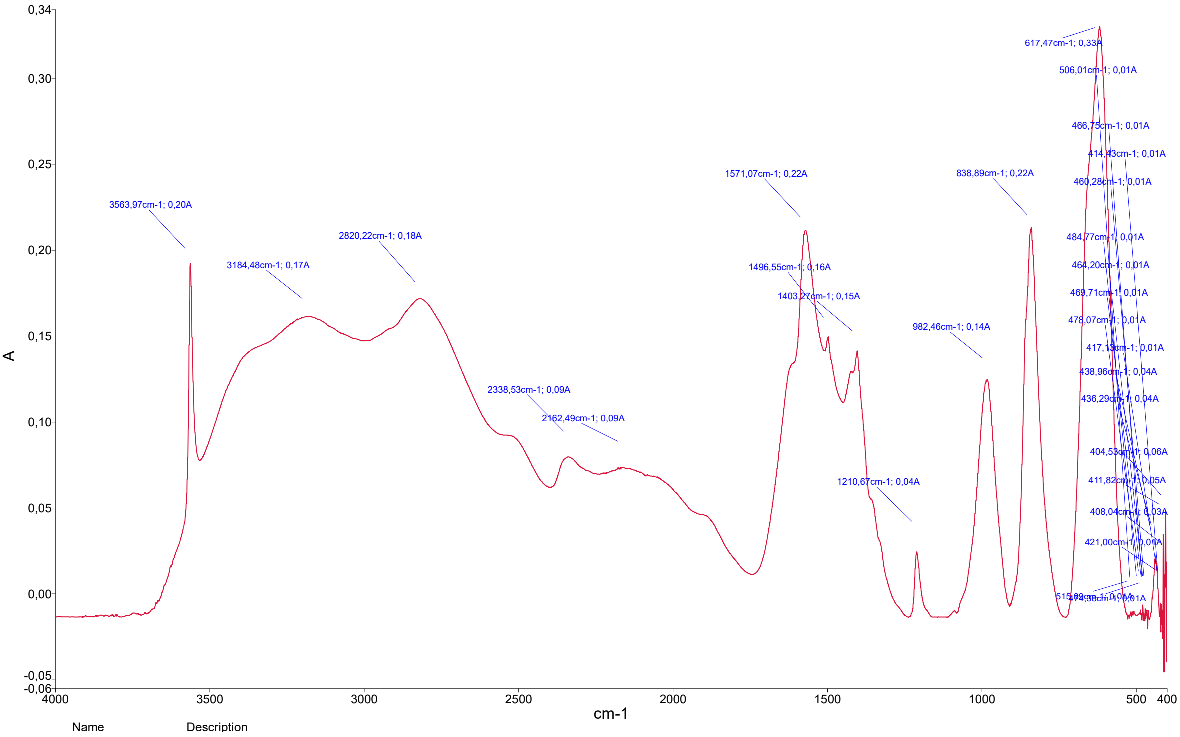

3. Dynamic light scattering

Table S1. Self-assembly of **3-6, 8, 9** in water by DLS method.

Sample	$C_{pillararene}$, μM	$d_{average}$, nm	PDI	ζ-potential, mV
**3**	1000	241±5	0.31±0.03	45±1.5
	100	268±17	0.41±0.02	-
	10	304±52	0.35±0.04	44.4
**4**	1000	400±154	0.47±0.12	-
	100	279±46	0.42±0.02	2.2±0.2
	10	-	-	-
**5**	1000	248±7	0.36±0.03	47±2
	100	239±14	0.40±0.06	-
	10	347±31	0.37±0.03	46.7 ±2
**6**	1000	459±113	0.62±0.06	-
	100	333±53	0.45±0.04	-
	10	214±23	0.49±0.03	3.5±2
**9**	100	141±5	0.27±0.01	17.5±2
	10	203±14	0.48±0.08	-
	1	158±39	0.31±0.03	-
**8**	100	112±5	0.38±0.10	3.1±0.7
	10	413±91	0.42±0.06	-
	1	296±85	0.46±0.09	-

Figure S33. Size distribution of the particles by intensity for **3** 1000 µM in water (PDI = 0.31± 0.03, d_{av} = 241± 5 nm).

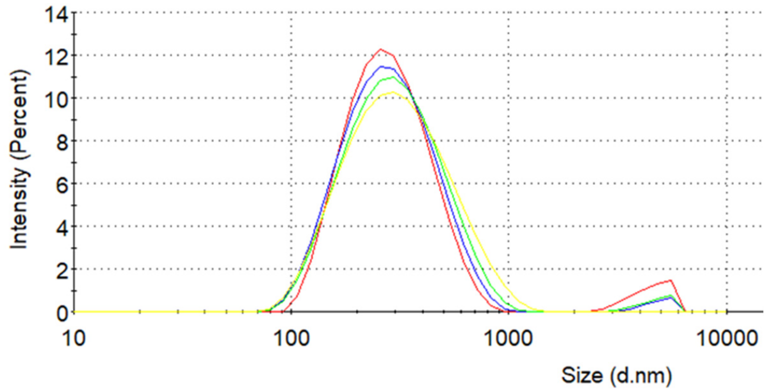

Figure S34. Size distribution of the particles by intensity for **5** 100 µM in water (PDI = 0.40±0.06, d_{av} = 239±14 nm).

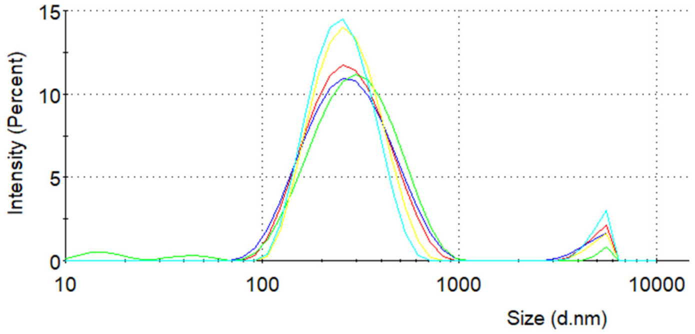

4. References

S1. Yao, Y.; Xue, M.; Chi, X.; Ma, Y.; He, J.; Abliz, Z.; Huang, F. A new water-soluble pillar[5]arene: synthesis and application in the preparation of gold nanoparticles. *Chem. Commun.* **2012,** *48*, 6505. 10.1039/c2cc31962d

S2. Mosmann, T. Rapid colorimetric assay for cellular growth and survival: application to proliferation and cytotoxicity assays. *J. Immunol. Methods.* **1983,** *65*, 55–63. 10.1016/0022-1759(83)90303-4

S3. Sarker S.D.; Nahar L.; Kumarasamy Y. Microtitre plate-based antibacterial assay incorporating resazurin as an indicator of cell growth, and its application in the in vitro antibacterial screening of phytochemicals. *Methods* **2007**, *42*, 321-324. 10.1016/j.ymeth.2007.01.006.

S4. Mortelmans K.; Zeiger E. The Ames Salmonella/microsome mutagenicity assay. *Mutat. Res.* **2000**, *455*, 29-60. 10.1016/s0027-5107(00)00064-6
